# Supplementary material for: Engineered reporter phages for detection of Escherichia coli, Enterococcus, and Klebsiella in urine
Source: Nat Commun. 2023 Jul 20;14:4336. doi: 10.1038/s41467-023-39863-x (PMC10359277; doi:10.1038/s41467-023-39863-x)
Supplement: Supplementary file 1 — Supplementary Information File [file 41467_2023_39863_MOESM1_ESM.pdf]

Supplementary Material – Meile et al. (2023)

Supplementary Figures

Figure S1. Species distribution analysis of the Zurich Uropathogen Collection, related to Fig. 1.

Figure S2. An *Enterococcus*-adapted pSelect CRISPR-Cas9 system programmed to restrict wild-type phage plaque formation while allowing propagation of recombinant phages, related to Fig. 2.

Figure S3. Reporter phage specificity analysis, activity in blood, and clonal diversity within the Zurich Uropathogen Collection, related to Fig. 4.

Figure S4. Species distribution analysis of strains from the field evaluation, related to Fig. 5.

Figure S5. Definition of test parameters for a reporter phage-based companion diagnostic, related to Fig. 6.

Supplementary Tables

Table S1. The Zurich Uropathogen Collection.

Table S2. Strains used in this study.

Table S3. Bacteria isolated during the field evaluation from patient urine.

Table S4: Plasmids used and constructed during this study.

Table S5: Primer and synthetic DNA string sequences used in this study.

Supplementary Figures

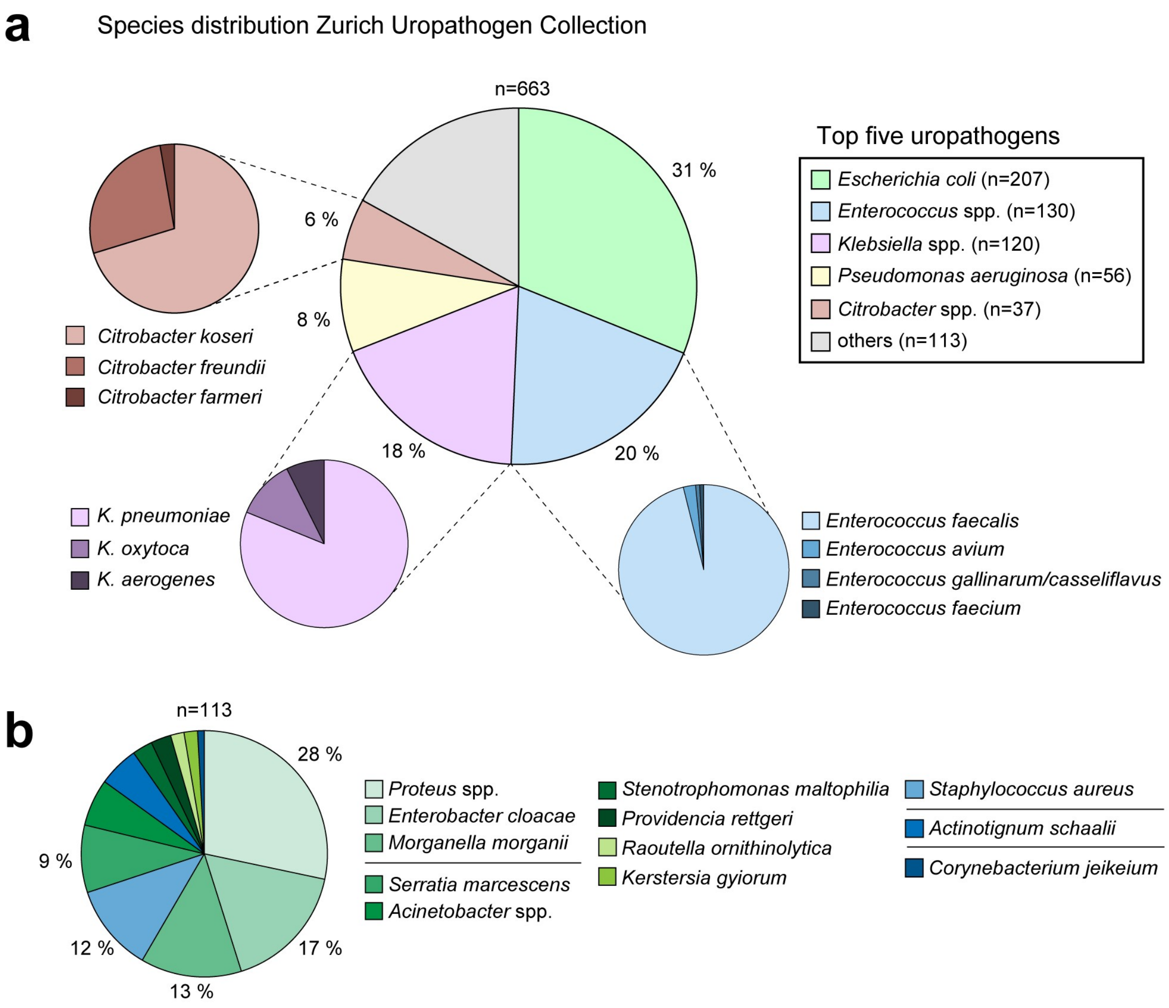

Figure S1. Species distribution analysis of the Zurich Uropathogen Collection, related to Fig. 1. (**a**) A total of 663 bacterial stains within the collection were analyzed with respect to their genus- and species distribution. Isolates that do not belong to the top five most prevalent uropathogens are shown in (**b**). *E. coli*, *Enterococcus* spp., and *Klebsiella* spp. isolates constitute the top three most prevalent uropathogens, collectively adding up to 69 % of isolates within the collection. Source data are provided as a Source Data file.

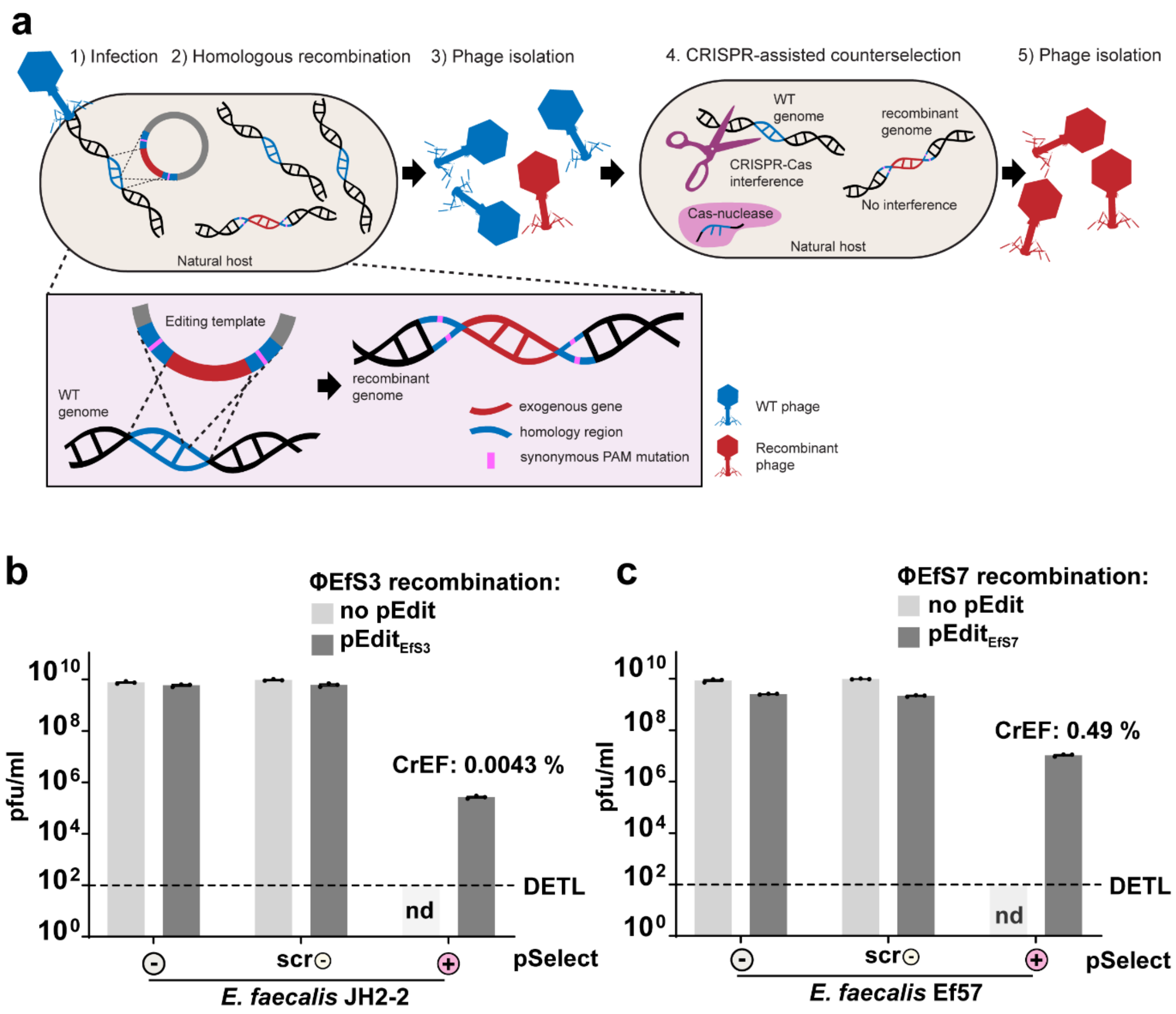

Figure S2. An *Enterococcus*-adapted pSelect CRISPR-Cas9 system programmed to restrict wild-type phage plaque formation while allowing propagation of recombinant phages, related to Fig. 2. (**a**) Schematic overview of the engineering workflow. (**b, c**) Phage lysates were generated on hosts without (light grey) or with (dark grey) editing plasmid (homologous recombination). Plaquing efficiency of (**b**) EfS3 lysates and (**c**) EfS7 lysates on hosts with (+) or without (-, scr) CRISPR targeting through the pSelect system. Data is mean +/- SEM of biological triplicates. As wildtype phages are fully restricted in the presence of pSelect, the CRISPR-escape frequency (PFU/mL_{+} / PFU/mL_{scr}) can be used as an approximation for the frequency of homologous recombination. scr = non-targeting crRNA; - = no pSelect; CrEF: CRISPR-escape frequency; DETL = detection limit for plaque formation; nd = not detected. Source data are provided as a Source Data file.

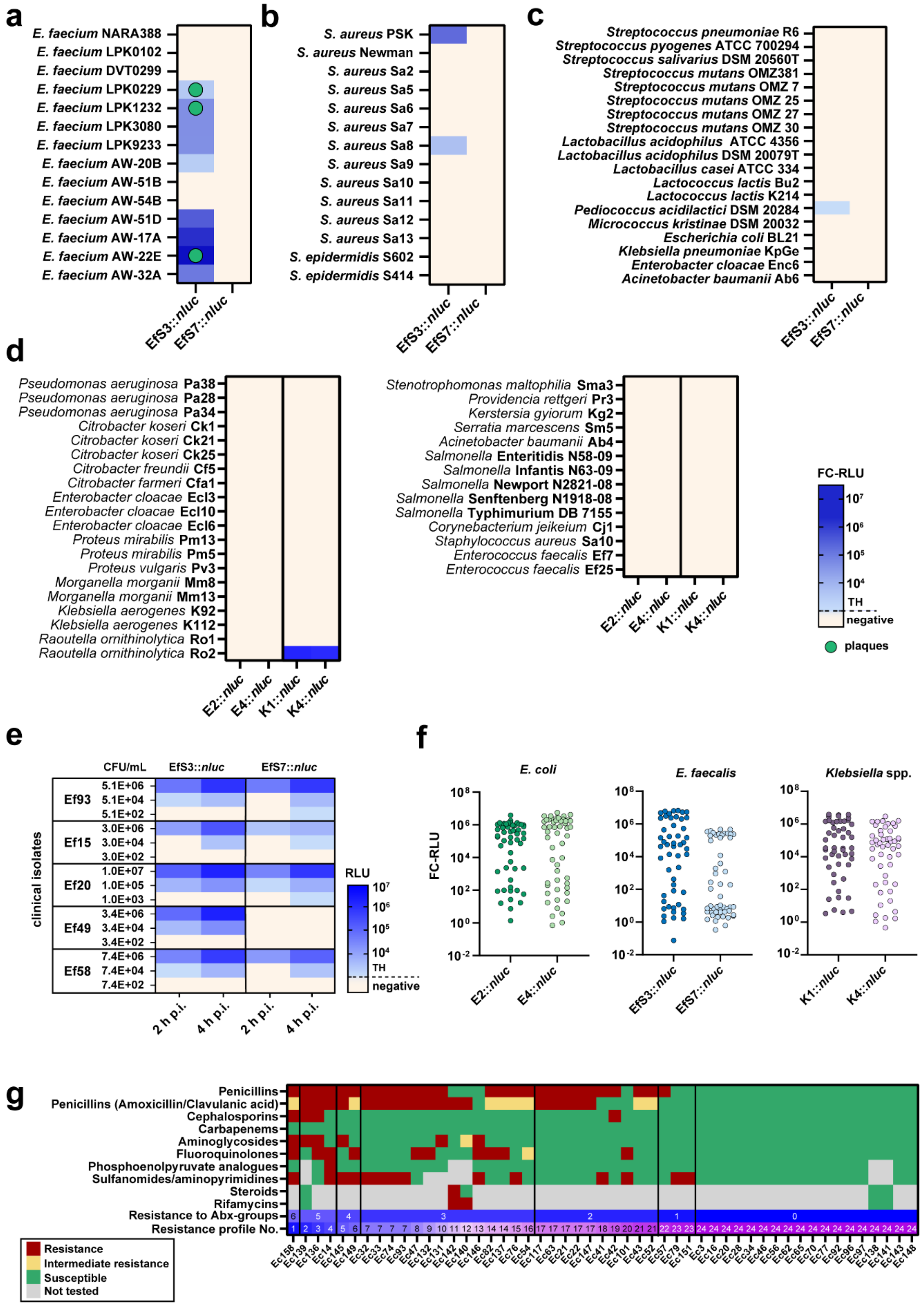

Figure S3. Reporter phage specificity analysis, activity in blood, and clonal diversity within the Zurich Uropathogen Collection, related to Fig. 4. To address phage specificity, the plaquing host ranges and bioluminescence detection ranges of phages EfS3*::nluc* and EfS7*::nluc* were determined on the indicated (**a**) *E. faecium* or (**b**) *S. aureus* strains and (**c**) on a set of additional Gram-positive and Gram-negative bacteria. (**d**) A similar specificity analysis was performed with reporter phages E2*::nluc*, E4*::nluc*, K1*::nluc*, and K4*::nluc*. The fold-change RLU (FC-RLU) is shown in shades of blue, plaque formation is indicated with a green dot. Thresholds (TH) are 50 in panel (a), (b) and (c) and 100 in panel (d). Values are mean from biological triplicates. (**e**) Heat map showing reporter phage-induced luminescence in human blood spiked with 6 selected

strains of *E. faecalis*. After 1/10 dilution of the spiked blood with buffered media and a 1h enrichment, reactions were mixed with $2x10^7$ PFU/mL *Enterococcus* reporter phages and RLU was determined at 2 h and 4 h p.i., TH is 10^3 RLU (EfS3) and $2.5x10^2$ (EfS7) (**f-g**) Analysis of the clonal diversity within the strain panel selected for host-range analysis. (**f**) To demonstrate strain diversity with respect to phage-sensitivity, the luminescence values (FC-RLU) from each reporter phage – host pair were plotted to visualize differences in signal production. Values are mean from biological triplicates. (**g**) To demonstrate strain diversity with respect to antibiotic sensitivity, all *E. coli* strains from the host range analysis were clustered according to their antibiotic resistance profiles. From 51 tested strains, 24 distinct patterns were identified, suggesting that E. *coli* clonal diversity is high. Source data are provided as a Source Data file.

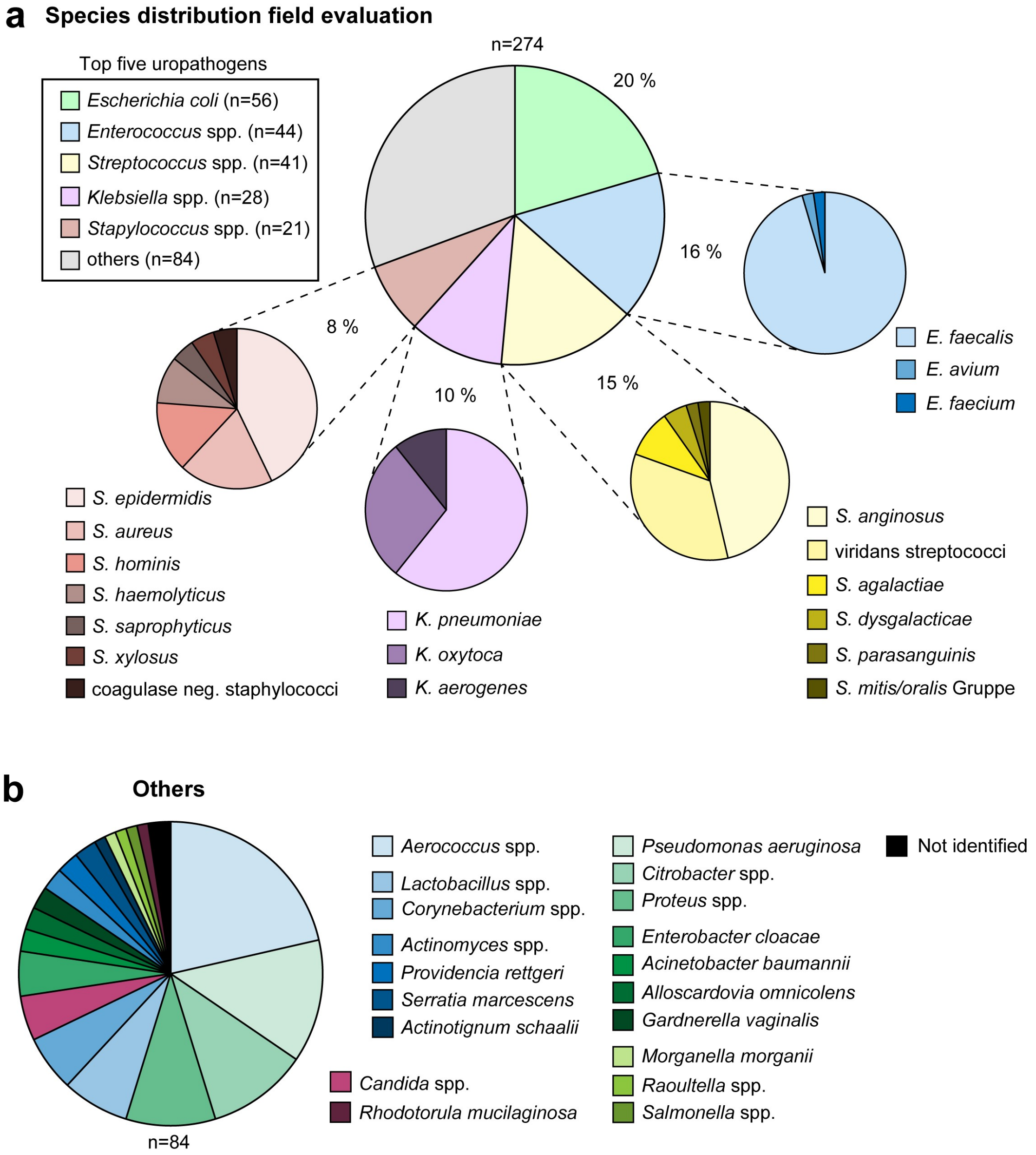

Figure S4. Species distribution analysis of strains from the field evaluation, related to Fig. 5. (**a**) A total of 274 bacterial stains identified during the field evaluation were analyzed with respect to their genus- and species distribution. Isolates that do not belong to the top five most prevalent uropathogens are shown in (**b**). Source data are provided as a Source Data file.

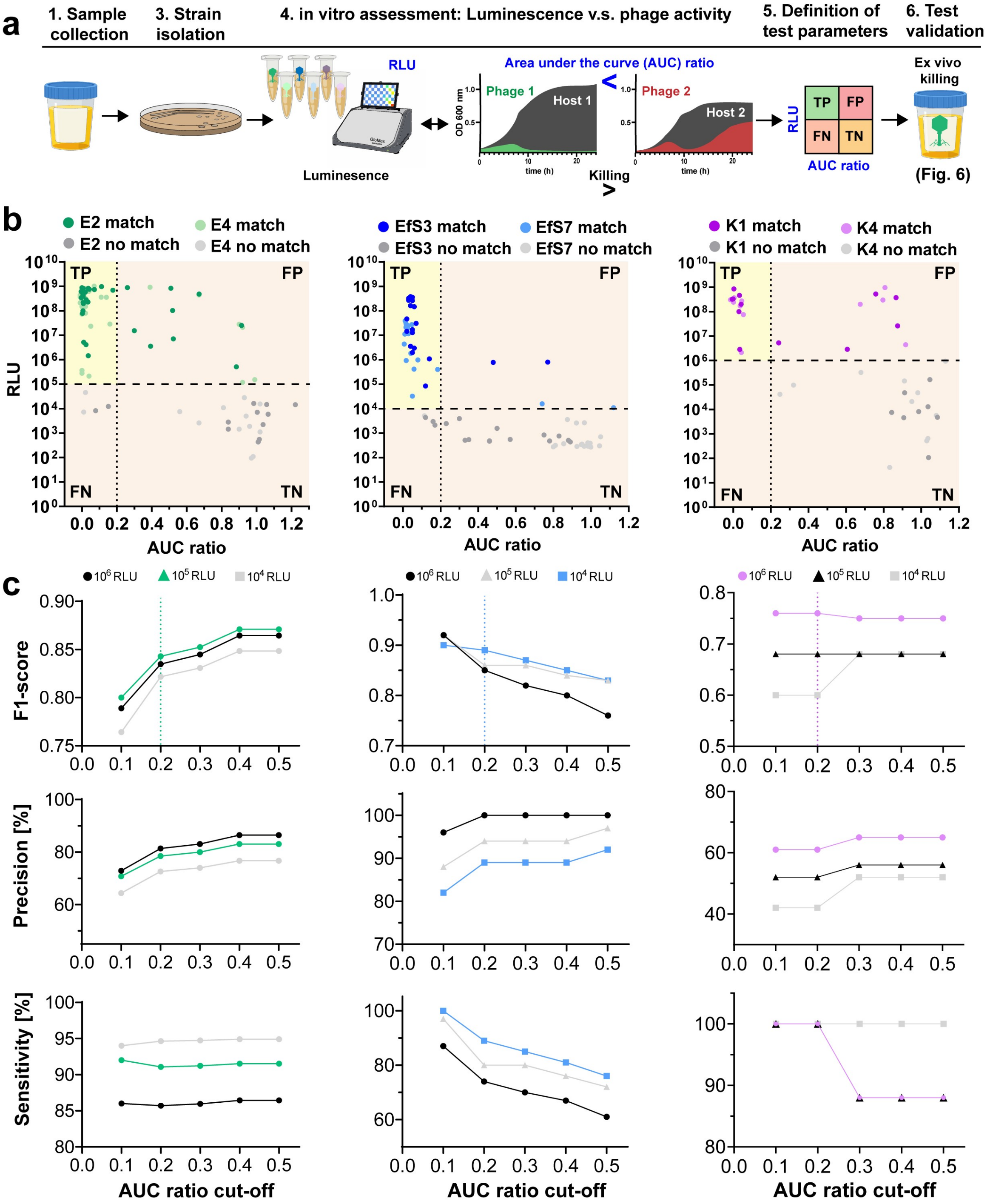

Figure S5. Definition of test parameters for a reporter phage-based companion diagnostic, related to Fig. 6. To develop a companion diagnostic assay that predicts phage antimicrobial activity, we correlated reporter phage-induced bioluminescence (RLU) with killing activity as determined by turbidity reduction assays (AUC ratio). Workflow shown in (**a**). The optimal RLU- and AUC ratio cutoffs were determined using defined experimental conditions. To this end, bacterial strains from the field evaluation were isolated and their luminescence response quantified using standardized infection conditions (starting phage titer = 10^7 PFU / ml; starting bacterial concentration = OD0.01). Activity was quantified as the ratio of the area under the curve (AUC ratio: $AUC_{HOST+PHAGE}$ / AUC_{HOST}) where a value of one corresponds to no activity and a value of zero corresponds to maximal activity. (**b**) For each phage-host pair, the AUC ratio was plotted against the raw luminescence value. (**c**) Test parameters that produce the most reliable prediction of success while maintaining a stringent activity cut-off were identified by comparison of F1-score (2*TP/(2*TP+FP+FN)), precision (TP/(TP+FP)), and sensitivity (TP/(TP+FN)) (see methods for more details). TP = true positives, TN = true negatives, FP = false positives, FN = false negatives. Parts of this figure were created with biorender.com. Source data are provided as a Source Data file.

Supplementary Tables

Table S1a. *E. coli* isolates of the Zurich Uropathogen Collection.

Species	Isolation month	UTI/ASB diagnosis	Isolate designation
Escherichia coli	January	ASB	Ec 1
Escherichia coli	January	UTI	Ec 3
Escherichia coli	January	ASB	Ec 4
Escherichia coli	January	ASB	Ec 6
Escherichia coli	January	ASB	Ec 10
Escherichia coli	January	ASB	Ec 13
Escherichia coli	January	ASB	Ec 12
Escherichia coli	January	ASB	Ec 9
Escherichia coli	January	UTI	Ec 11
Escherichia coli	January	ASB	Ec 5
Escherichia coli	January	ASB	Ec 2
Escherichia coli	January	ASB	Ec 18
Escherichia coli	January	ASB	Ec 17
Escherichia coli	January	UTI	Ec 16
Escherichia coli	January	ASB	Ec 15
Escherichia coli	January	UTI	Ec 14
Escherichia coli	January	ASB	Ec 8
Escherichia coli	January	ASB	Ec 7
Escherichia coli	January	ASB	Ec 19
Escherichia coli	January	ASB	Ec 27
Escherichia coli	January	ASB	Ec 26
Escherichia coli	January	ASB	Ec 24
Escherichia coli	January	ASB	Ec 23
Escherichia coli	January	UTI	Ec 21
Escherichia coli	January	ASB	Ec 168
Escherichia coli	January	ASB	Ec 169
Escherichia coli	January	UTI	Ec20
Escherichia coli	January	UTI	Ec 22
Escherichia coli	February	ASB	Ec 29
Escherichia coli	February	ASB	Ec 30
Escherichia coli	February	ASB	Ec 37
Escherichia coli	February	ASB	Ec 39
Escherichia coli	February	UTI	Ec 28
Escherichia coli	February	ASB	Ec 38
Escherichia coli	February	ASB	Ec 36
Escherichia coli	February	UTI	Ec 32
Escherichia coli	February	ASB	Ec 31
Escherichia coli	February	UTI	Ec 33
Escherichia coli	February	UTI	Ec 34
Escherichia coli	February	ASB	Ec 35
Escherichia coli	February	UTI	Ec 42
Escherichia coli	February	UTI	Ec 46
Escherichia coli	February	ASB	Ec 48
Escherichia coli	February	UTI	Ec 47
Escherichia coli	February	ASB	Ec 51
Escherichia coli	February	ASB	Ec 40
Escherichia coli	February	ASB	Ec 50
Escherichia coli	February	UTI	Ec 41
Escherichia coli	February	ASB	Ec 44
Escherichia coli	February	UTI	Ec 52
Escherichia coli	February	ASB	Ec 53
Escherichia coli	February	ASB	Ec 45
Escherichia coli	February	UTI	Ec 43
Escherichia coli	February	ASB	Ec 61

Escherichia coli	February	UTI	Ec 57
Escherichia coli	February	UTI	Ec 54
Escherichia coli	February	UTI	Ec 62
Escherichia coli	February	UTI	Ec 63
Escherichia coli	February	UTI	Ec 65
Escherichia coli	February	ASB	Ec 55
Escherichia coli	February	UTI	Ec 56
Escherichia coli	February	ASB	Ec 58
Escherichia coli	February	ASB	Ec 60
Escherichia coli	February	ASB	Ec 64
Escherichia coli	February	UTI	Ec 79
Escherichia coli	February	ASB	n/a
Escherichia coli	February	UTI	Ec 77
Escherichia coli	February	ASB	Ec 73
Escherichia coli	February	UTI	Ec 76
Escherichia coli	February	UTI	Ec 74
Escherichia coli	February	UTI	Ec 80
Escherichia coli	February	ASB	Ec 71
Escherichia coli	February	ASB	Ec 78
Escherichia coli	February	UTI	Ec 70
Escherichia coli	February	ASB	Ec 72
Escherichia coli	February	ASB	Ec 67
Escherichia coli	February	ASB	Ec 66
Escherichia coli	February	ASB	Ec 68
Escherichia coli	February	ASB	Ec 69
Escherichia coli	March	ASB	Ec 90
Escherichia coli	March	ASB	Ec 94
Escherichia coli	March	UTI	Ec 92
Escherichia coli	March	UTI	Ec 93
Escherichia coli	March	ASB	Ec 87
Escherichia coli	March	ASB	Ec 88
Escherichia coli	March	ASB	Ec 89
Escherichia coli	March	UTI	Ec 96
Escherichia coli	March	ASB	Ec 95
Escherichia coli	March	UTI	Ec 97
Escherichia coli	March	UTI	Ec 82
Escherichia coli	March	ASB	Ec 91
Escherichia coli	March	ASB	Ec222
Escherichia coli	March	ASB	Ec 84
Escherichia coli	March	UTI	Ec 83
Escherichia coli	March	ASB	Ec 85
Escherichia coli	March	UTI	Ec 86
Escherichia coli	March	ASB	Ec 107
Escherichia coli	March	ASB	Ec 103
Escherichia coli	March	ASB	Ec 102
Escherichia coli	March	UTI	Ec 104
Escherichia coli	March	ASB	Ec 105
Escherichia coli	March	ASB	Ec 100
Escherichia coli	March	ASB	Ec 99
Escherichia coli	March	ASB	Ec 98
Escherichia coli	March	ASB	Ec 108
Escherichia coli	March	ASB	Ec 106
Escherichia coli	March	ASB	Ec 109
Escherichia coli	March	ASB	Ec 110
Escherichia coli	March	UTI	Ec 101
Escherichia coli	March	ASB	Ec 135

Escherichia coli	March	ASB	Ec 126
Escherichia coli	March	ASB	Ec 128
Escherichia coli	March	UTI	Ec 129
Escherichia coli	March	ASB	Ec 130
Escherichia coli	March	UTI	Ec 131
Escherichia coli	April	UTI	Ec 132
Escherichia coli	April	ASB	Ec 133
Escherichia coli	April	UTI	Ec 134
Escherichia coli	April	ASB	Ec 111
Escherichia coli	April	ASB	Ec 112
Escherichia coli	April	ASB	Ec 113
Escherichia coli	April	ASB	Ec 114
Escherichia coli	April	ASB	Ec 115
Escherichia coli	April	ASB	Ec 116
Escherichia coli	April	UTI	Ec 117
Escherichia coli	April	ASB	Ec 118
Escherichia coli	April	ASB	Ec 119
Escherichia coli	April	ASB	Ec 120
Escherichia coli	April	ASB	Ec 121
Escherichia coli	April	ASB	Ec 122
Escherichia coli	April	ASB	Ec 123
Escherichia coli	April	ASB	Ec 124
Escherichia coli	April	ASB	Ec 125
Escherichia coli	April	ASB	n/a
Escherichia coli	April	UTI	Ec 136
Escherichia coli	April	UTI	Ec 138
Escherichia coli	April	UTI	Ec 142
Escherichia coli	April	UTI	Ec 137
Escherichia coli	April	UTI	Ec 139
Escherichia coli	April	UTI	Ec 141
Escherichia coli	May	UTI	Ec 140
Escherichia coli	May	UTI	Ec 143
Escherichia coli	May	UTI	Ec 145
Escherichia coli	May	UTI	Ec 146
Escherichia coli	May	UTI	Ec 147
Escherichia coli	May	UTI	Ec 148
Escherichia coli	May	UTI	Ec 149
Escherichia coli	May	UTI	Ec 151
Escherichia coli	May	UTI	Ec 150
Escherichia coli	May	UTI	Ec 153
Escherichia coli	May	UTI	Ec 152
Escherichia coli	June	UTI	Ec 154
Escherichia coli	June	UTI	Ec 155
Escherichia coli	June	UTI	Ec 156
Escherichia coli	June	UTI	Ec 158
Escherichia coli	June	UTI	Ec 159
Escherichia coli	June	UTI	Ec 157
Escherichia coli	July	UTI	Ec 160
Escherichia coli	July	UTI	Ec 161
Escherichia coli	July	UTI	Ec 162
Escherichia coli	July	UTI	Ec 167
Escherichia coli	July	UTI	Ec 165
Escherichia coli	July	UTI	Ec 166
Escherichia coli	August	ASB	Ec 171
Escherichia coli	August	UTI	Ec 170
Escherichia coli	August	UTI	Ec 164

Escherichia coli	August	UTI	Ec 163
Escherichia coli	August	UTI	Ec 173
Escherichia coli	August	UTI	Ec 174
Escherichia coli	August	UTI	Ec 172
Escherichia coli	August	UTI	Ec 178
Escherichia coli	August	UTI	Ec 175
Escherichia coli	September	UTI	Ec 176
Escherichia coli	September	UTI	Ec 177
Escherichia coli	September	UTI	Ec 179
Escherichia coli	September	UTI	Ec 182
Escherichia coli	October	UTI	Ec 180
Escherichia coli	October	UTI	Ec 181
Escherichia coli	October	UTI	Ec 184
Escherichia coli	October	UTI	Ec 185
Escherichia coli	October	UTI	Ec 186
Escherichia coli	October	UTI	Ec 187
Escherichia coli	October	UTI	Ec 188
Escherichia coli	October	UTI	Ec 189
Escherichia coli	October	UTI	Ec 190
Escherichia coli	October	UTI	Ec 192
Escherichia coli	October	UTI	Ec 193
Escherichia coli	November	UTI	Ec 194
Escherichia coli	November	UTI	Ec 195
Escherichia coli	November	UTI	Ec 198
Escherichia coli	November	UTI	Ec 200
Escherichia coli	November	UTI	Ec 202
Escherichia coli	November	UTI	Ec 204
Escherichia coli	November	UTI	Ec 210
Escherichia coli	November	UTI	Ec 208
Escherichia coli	November	UTI	Ec 209
Escherichia coli	November	UTI	Ec 211
Escherichia coli	November	UTI	Ec 212
Escherichia coli	November	UTI	Ec 221
Escherichia coli	November	UTI	Ec 213
Escherichia coli	December	UTI	Ec 214
Escherichia coli	December	UTI	Ec 215
Escherichia coli	December	UTI	Ec 216
Escherichia coli	December	UTI	Ec 217
Escherichia coli	December	UTI	Ec 218
Escherichia coli	December	UTI	Ec 219
Escherichia coli	December	UTI	Ec 220

Table S1b. *Klebsiella* spp. isolates of the Zurich Uropathogen Collection.

Species	Isolation month	UTI/ASB diagnosis	Isolate designation
Klebsiella oxytoca	January	ASB	Ko 2
Klebsiella pneumoniae	January	UTI	Kp 3
Klebsiella pneumoniae	January	ASB	Kp 8
Klebsiella oxytoca	January	ASB	Ko 7
Klebsiella pneumoniae	January	ASB	Kp 6
Klebsiella pneumoniae	January	ASB	Kp14
Klebsiella pneumoniae	January	ASB	Kp13
Klebsiella pneumoniae	January	ASB	Kp 11
Klebsiella pneumoniae	January	UTI	Kp 106
Klebsiella pneumoniae	January	UTI	Kp 5
Klebsiella pneumoniae	January	ASB	Kp 15
Klebsiella pneumoniae	January	ASB	Kp 9
Klebsiella pneumoniae	January	ASB	Kp 17
Klebsiella pneumoniae	January	ASB	Kp 18
Klebsiella pneumoniae	January	ASB	Kp 20
Klebsiella pneumoniae	January	UTI	Kp 16
Klebsiella pneumoniae	January	ASB	Kp 19
Klebsiella pneumoniae	February	ASB	Kp 25
Klebsiella pneumoniae	February	ASB	Kp 24
Klebsiella pneumoniae	February	ASB	Kp 23
Klebsiella pneumoniae	February	ASB	Kp 31
Klebsiella pneumoniae	February	ASB	Kp 30
Klebsiella pneumoniae	February	ASB	Kp 28
Klebsiella pneumoniae	February	UTI	Kp 26
Klebsiella pneumoniae	February	ASB	Kp 27
Klebsiella oxytoca	February	ASB	Ko 29
Klebsiella pneumoniae	February	ASB	Kp 22
Klebsiella pneumoniae	February	ASB	Kp 21
Klebsiella pneumoniae	February	UTI	Kp 36
Klebsiella pneumoniae	February	UTI	Kp 37
Klebsiella oxytoca	February	UTI	Ko 38
Klebsiella pneumoniae	February	UTI	Kp 34
Klebsiella pneumoniae	February	ASB	Kp 32
Klebsiella pneumoniae	February	ASB	Kp 41
Klebsiella pneumoniae	February	ASB	Kp 35
Klebsiella pneumoniae	February	ASB	Kp 40
Klebsiella pneumoniae	February	UTI	Kp 39
Klebsiella pneumoniae	February	UTI	Kp 43
Klebsiella pneumoniae	February	UTI	Kp 48
Klebsiella oxytoca	February	UTI	n/a
Klebsiella pneumoniae	February	UTI	Kp 45
Klebsiella pneumoniae	February	ASB	Kp 50
Klebsiella pneumoniae	February	ASB	Kp 52
Klebsiella oxytoca	February	ASB	Ko 49
Klebsiella (Enterobacter) aerogenes	February	ASB	Ka 44
Klebsiella pneumoniae	February	UTI	Kp 51
Klebsiella pneumoniae	February	ASB	Kp 46
Klebsiella pneumoniae	February	ASB	Kp 47
Klebsiella pneumoniae	March	ASB	Kp 61
Klebsiella pneumoniae	March	UTI	Kp 66
Klebsiella pneumoniae	March	ASB	Kp 60
Klebsiella oxytoca	March	ASB	Ko 63
Klebsiella pneumoniae	March	ASB	Kp 62
Klebsiella pneumoniae	March	ASB	Kp 55
Klebsiella pneumoniae	March	ASB	Kp 54
Klebsiella pneumoniae	March	ASB	Kp 59
Klebsiella pneumoniae	March	ASB	Kp 57

Klebsiella pneumoniae	March	ASB	Kp 56
Klebsiella pneumoniae	March	ASB	Kp 58
Klebsiella pneumoniae	March	ASB	Kp 64
Klebsiella (Enterobacter) aerogenes	March	ASB	Ka 65
Klebsiella pneumoniae	March	ASB	Kp 72
Klebsiella pneumoniae	March	ASB	Kp 70
Klebsiella pneumoniae	March	ASB	Kp 71
Klebsiella pneumoniae	March	ASB	Kp 74
Klebsiella pneumoniae	March	ASB	Kp 73
Klebsiella oxytoca	March	ASB	Ko 75
Klebsiella oxytoca	March	UTI	Ko 68
Klebsiella pneumoniae	March	UTI	Kp 69
Klebsiella pneumoniae	March	ASB	Kp 67
Klebsiella pneumoniae	March	ASB	Kp 82
Klebsiella pneumoniae	March	ASB	Kp 83
Klebsiella oxytoca	March	ASB	Ko 84
Klebsiella pneumoniae	March	ASB	Kp 85
Klebsiella pneumoniae	March	ASB	Kp 80
Klebsiella pneumoniae	March	ASB	Kp 81
Klebsiella pneumoniae	April	ASB	Kp 76
Klebsiella oxytoca	April	ASB	Ko 77
Klebsiella pneumoniae	April	ASB	Kp 78
Klebsiella pneumoniae	April	ASB	Kp 79
Klebsiella oxytoca	April	UTI	Ko 87
Klebsiella pneumoniae	April	UTI	Kp 93
Klebsiella pneumoniae	April	UTI	Kp 90
Klebsiella pneumoniae	April	UTI	Kp 89
Klebsiella pneumoniae	April	UTI	Kp 88
Klebsiella oxytoca	April	UTI	Ko 91
Klebsiella (Enterobacter) aerogenes	April	UTI	Ka 92
Klebsiella pneumoniae	May	UTI	Kp 94
Klebsiella pneumoniae	May	UTI	Kp 98
Klebsiella (Enterobacter) aerogenes	May	UTI	Ka 99
klebsiella pneumoniae	May	UTI	Kp 96
Klebsiella pneumoniae	May	UTI	Kp 95
Klebsiella pneumoniae	May	UTI	Kp 97
Klebsiella oxytoca	May	UTI	Ko 101
Klebsiella pneumoniae	May	UTI	Kp 100
Klebsiella pneumoniae	June	UTI	Kp 102
Klebsiella pneumoniae	June	UTI	Kp 103
Klebsiella pneumoniae	July	UTI	Kp 104
Klebsiella pneumoniae	July	UTI	Kp 105
Klebsiella pneumoniae	August	UTI	Kp 109
Klebsiella pneumoniae	August	UTI	Kp 108
Klebsiella pneumoniae	August	UTI	Kp 107
Klebsiella pneumoniae	August	UTI	Kp 110
Klebsiella (Enterobacter) aerogenes	August	UTI	Ka 114
Klebsiella pneumoniae	August	UTI	Kp 111
Klebsiella (Enterobacter) aerogenes	August	UTI	Ka 112
Klebsiella pneumoniae	September	UTI	Kp 113
Klebsiella (Enterobacter) aerogenes	September	UTI	Ka 119
Klebsiella (Enterobacter) aerogenes	September	UTI	Ka 115
Klebsiella pneumoniae	September	UTI	Kp 118
Klebsiella pneumoniae	September	UTI	Kp 120
Klebsiella pneumoniae	October	UTI	Kp 116
Klebsiella pneumoniae	October	UTI	Kp 117
Klebsiella pneumoniae	October	UTI	Kp 121
Klebsiella pneumoniae	October	UTI	Kp 112
Klebsiella pneumoniae	October	UTI	Kp 123

Klebsiella pneumoniae	October	UTI	Kp 124
Klebsiella pneumoniae	October	UTI	Kp 125
Klebsiella pneumoniae	November	UTI	Kp 126
Klebsiella pneumoniae	November	UTI	Kp 127

Table S1c. *Enterococcus* spp. isolates of the Zurich Uropathogen Collection.

Species	Isolation month	UTI/ASB diagnosis	Isolate designation
Enterococcus faecalis	January	ASB	Ef 8
Enterococcus faecalis	January	UTI	Ef 4
Enterococcus faecalis	January	ASB	Ef 1
Enterococcus faecalis	January	UTI	Ef 2
Enterococcus faecalis	January	ASB	Ef 3
Enterococcus faecalis	January	ASB	Ef 6
Enterococcus faecalis	January	ASB	Ef 5
Enterococcus faecalis	January	UTI	Ef 7
Enterococcus faecalis	February	ASB	Ef 15
Enterococcus faecalis	February	ASB	Ef 16
Enterococcus faecalis	February	ASB	Ef 18
Enterococcus faecalis	February	UTI	Ef 9
Enterococcus faecalis	February	ASB	Ef 10
Enterococcus faecalis	February	ASB	Ef 17
Enterococcus faecalis	February	UTI	Ec 12
Enterococcus faecalis	February	ASB	Ef 21
Enterococcus avium	February	UTI	Ea 1
Enterococcus faecalis	February	UTI	Ef 20
Enterococcus faecalis	February	ASB	Ef 11
Enterococcus faecalis	February	ASB	Ef 13
Enterococcus faecalis	February	ASB	Ef 14
Enterococcus faecalis	February	ASB	Ef 19
Enterococcus faecalis	February	ASB	Ef 24
Enterococcus faecalis	February	UTI	Ef 25
Enterococcus faecalis	February	ASB	Ef 28
Enterococcus faecalis	February	UTI	Ef 27
Enterococcus faecalis	February	ASB	Ef 22
Enterococcus faecalis	February	UTI	Ef 26
Enterococcus faecalis	February	ASB	Ef 23
Enterococcus faecalis	February	ASB	Ef 32
Enterococcus faecalis	February	UTI	Ef 34
Enterococcus faecalis	February	ASB	Ef 31
Enterococcus faecalis	February	UTI	n/a
Enterococcus faecalis	February	UTI	Ef 30
Enterococcus faecalis	February	ASB	Ef 29
Enterococcus faecalis	February	ASB	Ef 33
Enterococcus faecalis	February	ASB	Ef 37
Enterococcus faecalis	February	UTI	Ef 36
Enterococcus faecalis	February	ASB	Ef 42
Enterococcus faecalis	February	UTI	Ef 40
Enterococcus faecalis	February	ASB	Ef 43
Enterococcus faecalis	February	UTI	Ef 48
Enterococcus faecalis	February	UTI	Ef 47
Enterococcus faecalis	February	ASB	Ef 45
Enterococcus faecalis	February	ASB	Ef 44
Enterococcus faecalis	February	ASB	Ef 39
Enterococcus faecalis	February	ASB	Ef 46
Enterococcus faecalis	February	ASB	Ef 38
Enterococcus faecalis	March	ASB	Ef 61
Enterococcus faecalis	March	UTI	Ef 59
Enterococcus faecalis	March	UTI	Ef 57
Enterococcus faecalis	March	ASB	Ef 53
Enterococcus faecalis	March	ASB	Ef 60
Enterococcus faecalis	March	ASB	Ef 52
Enterococcus faecalis	March	UTI	Ef 58
Enterococcus faecalis	March	ASB	Ef 50

Enterococcus faecalis	March	ASB	Ef 49
Enterococcus faecalis	March	ASB	Ef 51
Enterococcus faecalis	March	ASB	Ef 62
Enterococcus faecalis	March	ASB	Ef 54
Enterococcus faecalis	March	ASB	Ef 55
Enterococcus faecalis	March	ASB	n/a
Enterococcus faecalis	March	ASB	n/a
Enterococcus faecalis	March	ASB	n/a
Enterococcus faecalis	March	ASB	Ef 64
Enterococcus faecalis	March	ASB	n/a
Enterococcus faecalis	March	ASB	Ef 63
Enterococcus faecalis	March	ASB	Ef 66
Enterococcus faecalis	March	UTI	Ef 65
Enterococcus faecalis	March	UTI	Ef 81
Enterococcus faecalis	March	ASB	Ef 82
Enterococcus faecalis	March	ASB	Ef 83
Enterococcus faecalis	March	ASB	Ef 84
Enterococcus faecalis	March	ASB	Ef85
Enterococcus faecalis	March	UTI	Ef 74
Enterococcus faecalis	March	ASB	Ef 76
Enterococcus faecalis	March	ASB	Ef 77
Enterococcus faecium	March	ASB	Efm 1
Enterococcus faecalis	March	ASB	Ef 78
Enterococcus faecalis	April	ASB	Ef 79
Enterococcus faecalis	April	ASB	Ef 80
Enterococcus faecalis	April	ASB	Ef67
Enterococcus faecalis	April	ASB	Ef 68
Enterococcus faecalis	April	ASB	Ef 69
Enterococcus faecalis	April	ASB	Ef 70
Enterococcus faecalis	April	ASB	Ef 71
Enterococcus faecalis	April	UTI	Ef 72
Enterococcus faecalis	April	ASB	Ef 73
Enterococcus faecalis	April	ASB	Ef 75
Enterococcus faecalis	April	UTI	Ef 90
Enterococcus faecalis	April	UTI	Ef 89
Enterococcus faecalis	April	UTI	Ef 91
Enterococcus faecalis	April	UTI	Ef 88
Enterococcus faecalis	May	UTI	Ef 94
Enterococcus faecalis	May	UTI	Ef 92
Enterococcus faecalis	May	UTI	Ef 93
Enterococcus faecalis	May	UTI	Ef 96
Enterococcus faecalis	June	UTI	Ef 95
Enterococcus faecalis	July	UTI	Ef 97
Enterococcus faecalis	July	UTI	Ef 98
Enterococcus faecalis	July	UTI	Ef 99
Enterococcus faecalis	July	UTI	Ef 100
Enterococcus faecalis	July	UTI	Ef 103
Enterococcus faecalis	August	UTI	Ef 109
Enterococcus faecalis	August	UTI	Ef 102
Enterococcus faecalis	August	UTI	Ef 101
Enterococcus faecalis	August	UTI	Ef 104
Enterococcus faecalis	August	UTI	Ef 105
Enterococcus faecalis	August	UTI	Ef 106
Enterococcus faecalis	August	UTI	Ef 107
Enterococcus faecalis	September	UTI	Ef 108
Enterococcus faecalis	September	UTI	Ef 110
Enterococcus faecalis	September	UTI	Ef 112
Enterococcus faecalis	October	UTI	Ef 111
Enterococcus avium	October	UTI	Ea 2

Enterococcus faecalis	October	UTI	Ef 113
Enterococcus faecalis	October	UTI	Ef 114
Enterococcus faecalis	October	UTI	Ef 115
Enterococcus faecalis	October	UTI	Ef 116
Enterococcus faecalis	October	UTI	Ef 117
Enterococcus gallinarum/casseliflavus	November	UTI	Egc 1
Enterococcus faecalis	November	UTI	Ef 118
Enterococcus avium	November	UTI	Ea 3
Enterococcus faecalis	November	UTI	Ef 129
Enterococcus faecalis	November	UTI	Ef 130
Enterococcus faecalis	December	UTI	Ef 131
Enterococcus faecalis	December	UTI	Ef 132
Enterococcus faecalis	December	UTI	Ef 133
Enterococcus faecalis	December	UTI	Ef 134
Enterococcus faecalis	November	UTI	Ef 128

Table S1d. Other species isolated as part of the Zurich Uropathogen Collection.			
**Species**	**Isolation month**	**UTI/ASB diagnosis**	**Isolate designation**
Citrobacter koseri (diversus)	January	ASB	Ck 1
Pseudomonas aeruginosa	January	ASB	Pa 1
Actinotignum (Actinobaculum) schaalii	January	ASB	n/a
Acinetobacter baumannii	January	UTI	Ab 4
Enterobacter cloacae	January	UTI	Enc 10
Proteus vulgaris/penneri	January	ASB	Pvp 1
Pseudomonas aeruginosa	January	ASB	Pa 3
Pseudomonas aeruginosa	January	ASB	Pa 2
Pseudomonas aeruginosa	January	ASB	Pa 38
Stenotrophomonas maltophilia	February	ASB	Sm 1
Staphylococcus aureus	February	ASB	Sa 1
Proteus mirabilis	February	UTI	Pm 1
Pseudomonas aeruginosa	February	ASB	Pa 4
Proteus mirabilis	February	ASB	Pm 3
Proteus vulgaris	February	ASB	Pv 1
Proteus mirabilis	February	UTI	Pm 2
Acinetobacter baumannii Komplex	February	ASB	Ab 1
Citrobacter koseri (diversus)	February	ASB	Ck 2
Pseudomonas aeruginosa	February	ASB	Pa 5
Pseudomonas aeruginosa	February	ASB	Pa 7
Morganella morganii	February	ASB	Mm 2
Proteus mirabilis	February	UTI	Pm 4
Morganella morganii	February	ASB	Mm 1
Pseudomonas aeruginosa	February	UTI	Pa 8
Citrobacter koseri (diversus)	February	ASB	Ck 4
Proteus mirabilis	February	UTI	Pm 6
Pseudomonas aeruginosa	February	ASB	Pa 6
Citrobacter freundii	February	ASB	Cf 1
Pseudomonas aeruginosa	February	ASB	Pa 9
Citrobacter freundii	February	ASB	Cf 2
Proteus mirabilis	February	UTI	Pm 5
Citrobacter koseri (diversus)	February	ASB	Ck 3
Serratia marcescens	February	ASB	Sms 1
Pseudomonas aeruginosa	February	ASB	Pa 10
Citrobacter koseri (diversus)	February	ASB	Ck 6
Citrobacter koseri (diversus)	February	ASB	Ck 5
Serratia marcescens	February	ASB	Sms 2
Proteus mirabilis	February	ASB	Pm 7
Proteus vulgaris/penneri	February	ASB	Pvp 2
Enterobacter cloacae	February	UTI	Enc 1.1
Enterobacter cloacae	February	ASB	Enc 2.1
Staphylococcus aureus	February	ASB	Sms 3
Serratia marcescens	February	ASB	Sa 3
Pseudomonas aeruginosa	February	UTI	Pa 13
Proteus vulgaris/penneri	February	UTI	Pvp 3
Staphylococcus aureus	February	ASB	Sa 4
Citrobacter koseri (diversus)	February	ASB	Ck 9
Proteus mirabilis	February	ASB	Pm 8
Proteus mirabilis	February	UTI	Pm 9
Pseudomonas aeruginosa	February	UTI	Pa 12
Acinetobacter baumannii	February	UTI	Ac 2
Staphylococcus aureus	February	ASB	Sa 2
Enterobacter cloacae	February	UTI	Enc 3.1
Morganella morganii	February	UTI	Mm 4
Morganella morganii	February	ASB	Mm 3
Citrobacter koseri (diversus)	February	ASB	Ck 10
Pseudomonas aeruginosa	February	UTI	Pa 11
Citrobacter koseri (diversus)	February	ASB	Ck 7
Citrobacter koseri (diversus)	February	ASB	Ck 8
Proteus mirabilis	March	ASB	Pm 11
Staphylococcus aureus	March	ASB	Sa 5
Actinotignum (Actinobaculum) schaalii	March	UTI	As 1
Pseudomonas aeruginosa	March	ASB	Pa 16

Providencia rettgeri	March	ASB	Pr 1
Proteus vulgaris/penneri	March	ASB	Pvp 6
Citrobacter koseri (diversus)	March	ASB	Ck 11
Pseudomonas aeruginosa	March	ASB	Pa 18
Staphylococcus aureus	March	ASB	Sa 6
Morganella morganii	March	ASB	Mm 6
Raoutella (Klebsiella) ornithinolytica	March	ASB	Ro 1
Pseudomonas aeruginosa	March	ASB	Pa 17
Citrobacter koseri (diversus)	March	ASB	Ck 13
Citrobacter freundii	March	ASB	Cf 3
Staphylococcus aureus	March	ASB	Sa 7
Proteus vulgaris/penneri	March	ASB	Pvp 5
Pseudomonas aeruginosa	March	ASB	Pa 15
Pseudomonas aeruginosa	March	ASB	Pa 19
Morganella morganii	March	ASB	Mm 5
Citrobacter koseri (diversus)	March	ASB	Ck 14
Pseudomonas aeruginosa	March	ASB	Pa 14
Citrobacter koseri (diversus)	March	ASB	Ck 12
Actinotignum (Actinobaculum) schaalii	March	ASB	As 2
Proteus mirabilis	March	ASB	Pm 10
Morganella morganii	March	ASB	Mm 7
Morganella morganii	March	ASB	Mm 8
Citrobacter koseri (diversus)	March	ASB	Ck 15
Morganella morganii	March	ASB	Mm 10
Pseudomonas aeruginosa	March	ASB	Pa 23
Citrobacter koseri (diversus)	March	ASB	Ck 17
Staphylococcus aureus	March	ASB	Sa 8
Citrobacter koseri (diversus)	March	ASB	Ck 16
Pseudomonas aeruginosa	March	ASB	Pa 24
Raoutella (Klebsiella) ornithinolytica	March	ASB	Ro 2
Serratia marcescens	March	UTI	Sms 5
Morganella morganii	March	UTI	Mm 9
Pseudomonas aeruginosa	March	ASB	Pa 21
Pseudomonas aeruginosa	March	ASB	Pa 22
Pseudomonas aeruginosa	March	ASB	Pa 20
Serratia marcescens	March	ASB	Sms 4
Serratia marcescens	March	ASB	Sms 7
Pseudomonas aeruginosa	March	ASB	Pa 31
Proteus vulgaris/penneri	March	ASB	Pvp 9
Staphylococcus aureus	March	ASB	Sa 10
Proteus mirabilis	March	ASB	Pm 13
Actinotignum (Actinobaculum) schaalii	March	ASB	As 3
Citrobacter koseri (diversus)	March	ASB	Ck 21
Pseudomonas aeruginosa	March	ASB	Pa 28
Pseudomonas aeruginosa	March	ASB	Pa 29
Pseudomonas aeruginosa	March	UTI	Pa 30
Acinetobacter baumannii Komplex	April	ASB	Ab 3
Stenotrophomonas maltophilia	April	ASB	Sm 2
Citrobacter koseri (diversus)	April	UTI	Ck 18
Pseudomonas aeruginosa	April	ASB	Pa 25
Serratia marcescens	April	ASB	Sms 6
Citrobacter koseri (diversus)	April	ASB	Ck 19
Citrobacter koseri (diversus)	April	ASB	Ck 20
Pseudomonas aeruginosa	April	ASB	Pa 26
Citrobacter freundii	April	ASB	Cf 4
Staphylococcus aureus	April	ASB	Sa 9
Providencia rettgeri	April	ASB	Pr 2
Kerstersia gyiorum	April	ASB	Kg 1
Proteus vulgaris/penneri	April	ASB	Pvp 7
Proteus vulgaris/penneri	April	ASB	Pvp 8
Pseudomonas aeruginosa	April	ASB	Pa 27
Morganella morganii	April	ASB	Mm 11
Proteus mirabilis	April	ASB	Pm 12
Pseudomonas aeruginosa	April	ASB	n/a
Citrobacter koseri (diversus)	April	UTI	Ck 22

Pseudomonas aeruginosa	April	UTI	Pa 32
Morganella morganii	April	UTI	Mm 12
Pseudomonas aeruginosa	April	UTI	Pa 33
Citrobacter koseri (diversus)	April	UTI	Ck 23
Morganella morganii	April	UTI	Mm 13
Enterobacter cloacae	April	UTI	Enc 3.2
Enterobacter cloacae	May	UTI	Enc 1.2
Citrobacter koseri (diversus)	May	UTI	Ck 24
Enterobacter cloacae	May	UTI	Enc 2.2
citrobacter koseri (diversus)	May	UTI	Ck 25
corynebacterium jeikeium	May	UTI	Cj 1
enterobacter cloacae	May	UTI	Enc 5
enterobacter cloacae	May	UTI	Enc 4
Pseudomonas aeruginosa	May	UTI	Pa 34
Proteus vulgaris	May	UTI	Pvp 10
Pseudomonas aeruginosa	June	UTI	Pa 35
Enterobacter cloacae	June	UTI	Enc 6
Pseudomonas aeruginosa	July	UTI	Pa 36
Serratia marcescens	July	UTI	Sms 8
Staphylococcus aureus	July	UTI	Sa 11
Proteus mirabilis	July	UTI	Pm 14
Enterobacter cloacae	July	UTI	Enc 12
Enterobacter cloacae	July	UTI	Enc 8
Enterobacter cloacae	July	UTI	Enc 13
Proteus mirabilis	August	UTI	Pm 16
Proteus vulgaris	August	UTI	Pvp 11
Proteus mirabilis	August	UTI	Pm 15
Pseudomonas aeruginosa	August	UTI	Pa 37
Citrobacter freundii	August	UTI	Cf 5
Pseudomonas aeruginosa	August	UTI	n/a
Pseudomonas aeruginosa	August	UTI	Pa 39
Proteus mirabilis	August	UTI	Pm 17
Pseudomonas aeruginosa	August	UTI	Pa 41
Pseudomonas aeruginosa	August	UTI	Pa 43
Acinetobacter ursingii	August	UTI	Au 1
Stenotrophomonas maltophilia	August	UTI	Sm 3
Citrobacter koseri (diversus)	August	UTI	Ck 27
Citrobacter freundii	August	UTI	Cf 6
Pseudomonas aeruginosa	August	UTI	Pa 42
Pseudomonas aeruginosa	August	UTI	Pa 44
Enterobacter cloacae	August	UTI	Enc 14
Pseudomonas aeruginosa	August	UTI	Pa 40
Proteus mirabilis	August	UTI	Pm 18
Staphylococcus aureus	August	UTI	Sa 12
Enterobacter cloacae	August	UTI	Enc 15
Acinetobacter baumannii	August	UTI	Ab 5
Serratia marcescens	August	UTI	Sms 9.1
Pseudomonas aeruginosa	August	UTI	Pa 45
Pseudomonas aeruginosa	Sepetember	UTI	Pa 46
Acinetobacter baumannii	Sepetember	UTI	Ab 6
Pseudomonas aeruginosa	Sepetember	UTI	Pa 48
Actinotignum (Actinobaculum) schaalii	October	UTI	As 4
Citrobacter freundii	October	UTI	Cf 7.1
Pseudomonas aeruginosa	October	UTI	Pa 47
Pseudomonas aeruginosa	October	UTI	Pa 49
Pseudomonas aeruginosa	October	UTI	Pa 50
Citrobacter freundii	October	UTI	Cf 8
Pseudomonas aeruginosa	October	UTI	Pa 51
Citrobacter freundii	October	UTI	Cf 9
Pseudomonas aeruginosa	October	UTI	Pa 52
Enterobacter cloacae	October	UTI	Enc 18
Proteus mirabilis	October	UTI	Pm 19
Morganella morganii	October	UTI	Mm 14
Pseudomonas aeruginosa	November	UTI	Pa 53
Enterobacter cloacae	November	UTI	Enc 19

Enterobacter cloacae	November	UTI	Enc 20
Staphylococcus aureus	November	UTI	Sa 13
Morganella morganii	November	UTI	Mm 15
Actinotignum (Actinobaculum) schaalii	November	UTI	As 5
Pseudomonas aeruginosa	November	UTI	Pa 54
Proteus vulgaris	November	UTI	Pvp 12
Proteus vulgaris	December	UTI	Pv 2
Providencia rettgeri	December	UTI	Pr 3
Kerstersia gyiorum	December	UTI	Kg 2
Serratia marcescens	December	UTI	Sms 9.2
Citrobacter farmeri	December	UTI	Cfa1
Citrobacter freundii	December	UTI	Cf 7.2
Enterobacter cloacae	August	UTI	Enc 16

Table S2. Strains used in this study. Sources: 1 = Zurich Uropathogen Collection; 2 = in house strain collection; 3= Collection of the Institute of Veterinary Bacteriology, University of Bern, Switzerland, 4= Wissing et. al.; 5= The National Reference Center for Emerging Antibiotic Resistance (NARA), University of Fribourg, Switzerland. 6= National Reference Centre for Enteropathogenic Bacteria and Listeria (NENT) (Zurich, Switzerland)

Escherichia coli			
**Bacterial species**	**Source**	**Designation**	**Application**
Escherichia coli	1	Ec3	Host range
Escherichia coli	1	Ec14	Host range
Escherichia coli	1	Ec16	Host range, phage isolation
Escherichia coli	1	Ec20	Host range
Escherichia coli	1	Ec21	Host range
Escherichia coli	1	Ec22	Host range
Escherichia coli	1	Ec28	Host range
Escherichia coli	1	Ec32	Host range
Escherichia coli	1	Ec33	Host range, phage isolation
Escherichia coli	1	Ec34	Host range
Escherichia coli	1	Ec41	Host range
Escherichia coli	1	Ec42	Host range, phage isolation
Escherichia coli	1	Ec43	Host range, phage isolation
Escherichia coli	1	Ec46	Host range
Escherichia coli	1	Ec47	Host range
Escherichia coli	1	Ec52	Host range
Escherichia coli	1	Ec54	Host range
Escherichia coli	1	Ec56	Host range, phage isolation
Escherichia coli	1	Ec57	Host range
Escherichia coli	1	Ec62	Host range
Escherichia coli	1	Ec63	Host range
Escherichia coli	1	Ec65	Host range, phage isolation
Escherichia coli	1	Ec70	Host range
Escherichia coli	1	Ec74	Host range
Escherichia coli	1	Ec76	Host range
Escherichia coli	1	Ec77	Host range
Escherichia coli	1	Ec79	Host range, phage isolation
Escherichia coli	1	Ec82	Host range
Escherichia coli	1	Ec92	Host range
Escherichia coli	1	Ec93	Host range
Escherichia coli	1	Ec96	Host range
Escherichia coli	1	Ec97	Host range
Escherichia coli	1	Ec101	Host range
Escherichia coli	1	Ec117	Host range
Escherichia coli	1	Ec131	Host range
Escherichia coli	1	Ec132	Host range
Escherichia coli	1	Ec136	Host range
Escherichia coli	1	Ec137	Host range
Escherichia coli	1	Ec138	Host range
Escherichia coli	1	Ec139	Host range
Escherichia coli	1	Ec140	Host range
Escherichia coli	1	Ec141	Host range
Escherichia coli	1	Ec142	Host range
Escherichia coli	1	Ec143	Host range
Escherichia coli	1	Ec145	Host range
Escherichia coli	1	Ec146	Host range
Escherichia coli	1	Ec147	Host range
Escherichia coli	1	Ec148	Host range
Escherichia coli	1	Ec149	Host range
Escherichia coli	1	Ec151	Host range
Escherichia coli	1	Ec158	Host range
Escherichia coli	2	BL21	Host range, phage isolation, propagation

***Klebsiella* spp.**			
**Bacterial species**	**Source**	**Designation**	**Application**
Klebsiella aerogenes	1	k92	Host range
Klebsiella aerogenes	1	k112	Host range
Klebsiella oxytoca	1	Ko38	Host range, phage isolation
Klebsiella oxytoca	1	Ko68	Host range
Klebsiella oxytoca	1	Ko87	Host range
Klebsiella oxytoca	1	Ko101	Host range
Klebsiella pneumoniae	1	KpGe (Kp1)	Host range, phage isolation, propagation
Klebsiella pneumoniae	1	Kp3	Host range, phage isolation
Klebsiella pneumoniae	1	Kp5	Host range
Klebsiella pneumoniae	1	Kp6	Host range
Klebsiella pneumoniae	1	Kp8	Host range
Klebsiella pneumoniae	1	Kp11	Host range
Klebsiella pneumoniae	1	Kp13	Host range
Klebsiella pneumoniae	1	Kp14	Host range
Klebsiella pneumoniae	1	Kp15	Host range
Klebsiella pneumoniae	1	Kp16	Host range
Klebsiella pneumoniae	1	Kp21	Host range
Klebsiella pneumoniae	1	Kp22	Host range, phage isolation
Klebsiella pneumoniae	1	Kp26	Host range
Klebsiella pneumoniae	1	Kp31	Host range, phage isolation
Klebsiella pneumoniae	1	Kp33	Host range
Klebsiella pneumoniae	1	Kp34	Host range
Klebsiella pneumoniae	1	Kp36	Host range, phage isolation
Klebsiella pneumoniae	1	Kp37	Host range
Klebsiella pneumoniae	1	Kp39	Host range, phage isolation
Klebsiella pneumoniae	1	Kp43	Host range, phage isolation
Klebsiella pneumoniae	1	Kp45	Host range
Klebsiella pneumoniae	1	Kp48	Host range
Klebsiella pneumoniae	1	Kp51	Host range
Klebsiella pneumoniae	1	Kp59	Host range
Klebsiella pneumoniae	1	Kp66	Host range
Klebsiella pneumoniae	1	Kp67	Host range
Klebsiella pneumoniae	1	Kp69	Host range
Klebsiella pneumoniae	1	Kp70	Host range
Klebsiella pneumoniae	1	Kp71	Host range
Klebsiella pneumoniae	1	Kp72	Host range
Klebsiella pneumoniae	1	Kp76	Host range
Klebsiella pneumoniae	1	Kp78	Host range
Klebsiella pneumoniae	1	Kp79	Host range
Klebsiella pneumoniae	1	Kp80	Host range
Klebsiella pneumoniae	1	Kp81	Host range
Klebsiella pneumoniae	1	Kp82	Host range
Klebsiella pneumoniae	1	Kp83	Host range
Klebsiella pneumoniae	1	Kp88	Host range
Klebsiella pneumoniae	1	Kp89	Host range
Klebsiella pneumoniae	1	Kp90	Host range
Klebsiella pneumoniae	1	Kp93	Host range
Klebsiella pneumoniae	1	Kp94	Host range
Klebsiella pneumoniae	1	Kp95	Host range
Klebsiella pneumoniae	1	Kp96	Host range
Klebsiella pneumoniae	1	Kp97	Host range
Klebsiella pneumoniae	1	Kp98	Host range

***Enterococcus* spp.**			
**Bacterial species**	**Source**	**Designation**	**Application**
Enterococcus faecalis	1	Ef2	Host range
Enterococcus faecalis	1	Ef3	Host range, phage isolation
Enterococcus faecalis	1	Ef4	Host range
Enterococcus faecalis	1	Ef5	Host range
Enterococcus faecalis	1	Ef7	Host range
Enterococcus faecalis	1	Ef8	Phage isolation
Enterococcus faecalis	1	Ef12	Host range
Enterococcus faecalis	1	Ef14	Phage isolation
Enterococcus faecalis	1	Ef15	Host range
Enterococcus faecalis	1	Ef18	Host range
Enterococcus faecalis	1	Ef20	Host range
Enterococcus faecalis	1	Ef24	Host range
Enterococcus faecalis	1	Ef25	Host range
Enterococcus faecalis	1	Ef26	Host range
Enterococcus faecalis	1	Ef27	Host range
Enterococcus faecalis	1	Ef28	Host range
Enterococcus faecalis	1	Ef30	Host range
Enterococcus faecalis	1	Ef33	phage isolation
Enterococcus faecalis	1	Ef34	Host range
Enterococcus faecalis	1	Ef36	Host range, phage isolation
Enterococcus faecalis	1	Ef38	Host range
Enterococcus faecalis	1	Ef44	Host range
Enterococcus faecalis	1	Ef45	Host range
Enterococcus faecalis	1	Ef46	Host range
Enterococcus faecalis	1	Ef47	Host range
Enterococcus faecalis	1	Ef48	Host range
Enterococcus faecalis	1	Ef49	Host range
Enterococcus faecalis	1	Ef51	Host range, phage isolation
Enterococcus faecalis	1	Ef54	Host range, phage isolation
Enterococcus faecalis	1	Ef55	Host range
Enterococcus faecalis	1	Ef57	Host range, phage isolation, propagation
Enterococcus faecalis	1	Ef58	Host range
Enterococcus faecalis	1	Ef59	Host range
Enterococcus faecalis	1	Ef60	Host range
Enterococcus faecalis	1	Ef66	Host range
Enterococcus faecalis	1	Ef67	Host range
Enterococcus faecalis	1	Ef69	Host range
Enterococcus faecalis	1	Ef70	Host range
Enterococcus faecalis	1	Ef71	Host range
Enterococcus faecalis	1	Ef72	Host range
Enterococcus faecalis	1	Ef73	Host range
Enterococcus faecalis	1	Ef74	Host range
Enterococcus faecalis	1	Ef78	Host range, phage isolation
Enterococcus faecalis	1	Ef79	Host range
Enterococcus faecalis	1	Ef81	Host range
Enterococcus faecalis	1	Ef82	Host range
Enterococcus faecalis	1	Ef83	Host range
Enterococcus faecalis	1	Ef88	Host range
Enterococcus faecalis	1	Ef89	Host range
Enterococcus faecalis	1	Ef90	Host range
Enterococcus faecalis	1	Ef92	Host range
Enterococcus faecalis	1	Ef93	Host range
Enterococcus faecalis	1	Ef94	Host range
Enterococcus faecalis	1	Ef95	Host range
Enterococcus faecalis	1	Ef96	Host range, phage isolation
Enterococcus faecalis	2	JH2-2	phage isolation, propagation
Enterococcus faecium	5	NARA388	Specificity testing
Enterococcus faecium	3	LPK0102	Specificity testing
Enterococcus faecium	3	DVT02299	Specificity testing
Enterococcus faecium	3	LPK0229	Specificity testing
Enterococcus faecium	3	LPK1232	Specificity testing
Enterococcus faecium	3	LPK3080	Specificity testing
Enterococcus faecium	3	LPK9233	Specificity testing
Enterococcus faecium	4	AW-51B	Specificity testing
Enterococcus faecium	4	AW-54B	Specificity testing
Enterococcus faecium	4	AW-51D	Specificity testing
Enterococcus faecium	4	AW-17A	Specificity testing
Enterococcus faecium	4	AW-22E	Specificity testing
Enterococcus faecium	4	AW-32A	Specificity testing

**Other Gram-positives**			
**Bacterial species**	**Source**	**Designation**	**Application**
Corynebacterium jeikeium	1	Cj1	Specificity testing
Lactobacillus acidophilus	2	ATCC4356	Specificity testing
Lactobacillus acidophilus	2	DSM20079T	Specificity testing
Lactobacillus casei	2	ATCC334	Specificity testing
Lactococcus lactis	2	Bu2	Specificity testing
Lactococcus lactis	2	K214	Specificity testing
Micrococcus kristinae	2	DSM20032	Specificity testing
Pediococcus acidilactici	2	DSM20284	Specificity testing
Staphylococcus aureus	2	Newman	Specificity testing
Staphylococcus aureus	1	Sa2	Specificity testing
Staphylococcus aureus	1	Sa5	Specificity testing
Staphylococcus aureus	1	Sa6	Specificity testing
Staphylococcus aureus	1	Sa7	Specificity testing
Staphylococcus aureus	1	Sa8	Specificity testing
Staphylococcus aureus	1	Sa9	Specificity testing
Staphylococcus aureus	1	Sa10	Specificity testing
Staphylococcus aureus	1	Sa11	Specificity testing
Staphylococcus aureus	1	Sa12	Specificity testing
Staphylococcus aureus	1	Sa13	Specificity testing
Staphylococcus aureus	2	PSK	Specificity testing
Staphylococcus epidermidis	2	S602	Specificity testing
Staphylococcus epidermidis	2	S414	Specificity testing
Streptococcus mutans	2	OMZ381	Specificity testing
Streptococcus mutans	2	OMZ7	Specificity testing
Streptococcus mutans	2	OMZ25	Specificity testing
Streptococcus mutans	2	OMZ27	Specificity testing
Streptococcus mutans	2	OMZ30	Specificity testing
Streptococcus pneumoniae	2	R6	Specificity testing
Streptococcus pyogenes	2	ATCC700294	Specificity testing
Streptococcus salivarius	2	DSM20560T	Specificity testing

**Other Gram-negatives**			
**Bacterial species**	**Source**	**Designation**	**Application**
Acinetobacter baumanii	1	Ab4	Specificity testing
Acinetobacter baumanii	1	Ab6	Specificity testing
Citrobacter farmeri	1	Cfa1	Specificity testing
Citrobacter freundii	1	Cf5	Specificity testing
Citrobacter koseri	1	Ck1	Specificity testing
Citrobacter koseri	1	Ck21	Specificity testing
Citrobacter koseri	1	Ck25	Specificity testing
Enterobacter cloacae	1	Ecl3	Specificity testing
Enterobacter cloacae	1	Ecl10	Specificity testing
Enterobacter cloacae	1	Ecl6	Specificity testing
Enterobacter cloacae	1	Enc6	Specificity testing
Kerstersia gyiorum	1	Kg2	Specificity testing
Morganella morganii	1	Mm8	Specificity testing
Morganella morganii	1	Mm13	Specificity testing
Proteus mirabilis	1	Pm13	Specificity testing
Proteus mirabilis	1	Pm5	Specificity testing
Proteus vulgaris	1	Pv3	Specificity testing
Providencia rettgeri	1	Pr3	Specificity testing
Pseudomonas aeruginosa	1	Pa38	Specificity testing
Pseudomonas aeruginosa	1	Pa28	Specificity testing
Pseudomonas aeruginosa	1	Pa34	Specificity testing
Raoutella ornithinolytica	1	Ro1	Specificity testing
Raoutella ornithinolytica	1	Ro2	Specificity testing
Salmonella Enteritidis	6	N58-09	Specificity testing
Salmonella Infantis	6	N63-09	Specificity testing
Salmonella Newport	6	N2821-08	Specificity testing
Salmonella Senftenberg	6	N1918-08	Specificity testing
Salmonella Typhimurium	2	DB 7155	Specificity testing
Serratia marcescens	1	Sm5	Specificity testing
Stenotrophomonas maltophilia	1	Sma3	Specificity testing

Table S3: Bacteria isolated during the field evaluation from patient urine. The source of the urine corresponds to the patient number. Nr 1-147: Site 1 (Balgrist University Hospital, Zurich, Switzerland); Nr. 148-206, Site 2 (University Hospital Zurich, Zurich, Switzerland).

Escherichia coli		
**Species designation**	**Strain designation**	**Patient Nr. field evaluation**
Eschericia coli	BAL Ec1	6
Eschericia coli	BAL Ec2	7
Eschericia coli	BAL Ec3	8
Eschericia coli	BAL Ec4	13
Eschericia coli	BAL Ec5	14
Eschericia coli	BAL Ec6	17
Eschericia coli	BAL Ec7	19
Eschericia coli	BAL Ec8	23
Eschericia coli	BAL Ec9	24
Eschericia coli	BAL Ec10	26
Eschericia coli	BAL Ec11	35
Eschericia coli	BAL Ec12	41
Eschericia coli	BAL Ec13	43
Eschericia coli	BAL Ec14	45
Eschericia coli	BAL Ec15	48
Eschericia coli	BAL Ec16	53
Eschericia coli	BAL Ec17	56
Eschericia coli	BAL Ec18	57
Eschericia coli	BAL Ec19	64
Eschericia coli	BAL Ec20	75
Eschericia coli	BAL Ec21	78
Eschericia coli	BAL Ec22	79
Eschericia coli	BAL Ec23	80
Eschericia coli	BAL Ec24	86
Eschericia coli	BAL Ec25	88
Eschericia coli	BAL Ec26	93
Eschericia coli	BAL Ec27	108
Eschericia coli	BAL Ec28	111
Eschericia coli	BAL Ec29	115
Eschericia coli	BAL Ec30	119
Eschericia coli	BAL Ec31	128
Eschericia coli	BAL Ec32	133
Eschericia coli	BAL Ec33	134
Eschericia coli	BAL Ec34	135
Eschericia coli	BAL Ec35	141
Eschericia coli	USZ Ec01	148
Eschericia coli	USZ Ec03	150
Eschericia coli	USZ Ec07	154
Eschericia coli	USZ Ec12	159
Eschericia coli	USZ Ec14	161
Eschericia coli	USZ Ec24	171
Eschericia coli	USZ Ec33	180
Eschericia coli	USZ Ec38	185
Eschericia coli	USZ Ec39	186
Eschericia coli	USZ Ec40	187
Eschericia coli	USZ Ec41	188
Eschericia coli	USZ Ec42	189
Eschericia coli	USZ Ec53	200
Eschericia coli	USZ Ec56	203
Eschericia coli	USZ Ec59	206

***Klebsiella* spp.**		
**Species complex designation**	**Strain designation**	**Patient Nr. field evaluation**
Klebsiella aerogenes	USZ Ka11	158
Klebsiella aerogenes	USZ Ka53	200
Klebsiella oxytoca	BAL Ko1	27
Klebsiella oxytoca	BAL Ko2	48
Klebsiella oxytoca	BAL Ko3	65
Klebsiella oxytoca	BAL Ko4	139
Klebsiella oxytoca	USZ Ko09	156
Klebsiella oxytoca	USZ Ko51	198
Klebsiella pneumoniae	BAL Kp1	11
Klebsiella pneumoniae	BAL Kp2	26
Klebsiella pneumoniae	BAL Kp5	76
Klebsiella pneumoniae	BAL Kp6	77
Klebsiella pneumoniae	BAL Kp7	84
Klebsiella pneumoniae	BAL Kp8	87
Klebsiella pneumoniae	BAL Kp9	117
Klebsiella pneumoniae	BAL Kp10	118
Klebsiella pneumoniae	BAL Kp11	143
Klebsiella pneumoniae	BAL Kp12	146
Klebsiella pneumoniae	USZ Kp08	155
Klebsiella pneumoniae	USZ Kp37	184
Klebsiella pneumoniae	USZ Kp38	185
Klebsiella pneumoniae	USZ Kp41	188

***Enterococcus* spp.**		
**Species designation**	**Strain designation**	**Patient Nr. field evaluation**
Enterococcus avium	USZ Ea08	155
Enterococcus faecalis	BAL Ef1	1
Enterococcus faecalis	BAL Ef2	7
Enterococcus faecalis	BAL Ef3	36
Enterococcus faecalis	BAL Ef4	41
Enterococcus faecalis	BAL Ef5	43
Enterococcus faecalis	BAL Ef6	57
Enterococcus faecalis	BAL Ef7	63
Enterococcus faecalis	BAL Ef8	76
Enterococcus faecalis	BAL Ef9	89
Enterococcus faecalis	BAL Ef10	96
Enterococcus faecalis	BAL Ef19	107
Enterococcus faecalis	BAL Ef11	110
Enterococcus faecalis	BAL Ef12	113
Enterococcus faecalis	BAL Ef13	119
Enterococcus faecalis	BAL Ef14	126
Enterococcus faecalis	BAL Ef15	129
Enterococcus faecalis	BAL Ef16	131
Enterococcus faecalis	BAL Ef17	139
Enterococcus faecalis	BAL Ef18	144
Enterococcus faecalis	USZ Ef08	155
Enterococcus faecalis	USZ Ef09	156
Enterococcus faecalis	USZ Ef14	161
Enterococcus faecalis	USZ Ef15	162
Enterococcus faecalis	USZ Ef16	163
Enterococcus faecalis	USZ Ef28	175
Enterococcus faecalis	USZ Ef32	179
Enterococcus faecalis	USZ Ef33	180
Enterococcus faecalis	USZ Ef38	185
Enterococcus faecalis	USZ Ef39	186
Enterococcus faecalis	USZ Ef40	187
Enterococcus faecalis	USZ Ef41	188
Enterococcus faecalis	USZ Ef43	190
Enterococcus faecalis	USZ Ef46	193
Enterococcus faecalis	USZ Ef48	195
Enterococcus faecalis	USZ Ef56	203
Enterococcus faecalis	USZ Ef59	206

Table S4: Plasmids used and constructed during this study. P1 = Primer 1, P2 = Primer 2, *cps* = major capsid protein gene, *nluc* = nanoluciferase gene.

Name	Fragment	Template	P1	P2	Reference	Application
pLEB579	-<br>-	-<br>-	-<br>-	-<br>-	Beasley et al., 2004	backbone Gram-positives
pCas9	-<br>-	-<br>-	-<br>-	-<br>-	Jiang et al., 2013	backbone Gram-negatives
pUC19	-<br>-	-<br>-	-<br>-	-<br>-	Yanisch-Perron et al., 1985	backbone Gram-negatives
pRSF-deut1	-<br>-	-<br>-	-<br>-	-<br>-	Novagen, Merck	backbone Gram-negatives
pUC19-Kan^{R}	F1<br>F2	pRSF-deut1<br>pUC19	P37<br>P39	P38<br>P40	this study	backbone Gram-negatives
pCas9$_{T4}$	F1<br>F2	pCas9<br>String$_{T4\text{-}RSRSR}$	P5<br>P7	P6<br>P8	this study	spacer pair targeting *cps* of phage T4, scrambled (scr) control vector in Gram-negatives
pSelect$_{E}$	F1<br>F2	pCas9<br>String$_{E\text{-}RSRSR}$	P5<br>P7	P6<br>P8	this study	spacer pair targeting *cps* and *gp170* of phage E2/E4
pSelect$_{K}$	F1<br>F2	pCas9<br>String$_{K\text{-}RSRSR}$	P5<br>P7	P6<br>P8	this study	spacer pair targeting *gp167* and *gp168* of phage K1 (= spacer pair targeting *gp168* and *gp169* of phage K4)
pSelect$_{T4}$	F1<br>F2	pLEB579<br>pCas9$_{T4}$	P1<br>P3	P2<br>P4	this study	spacer pair targeting *cps* of phage T4, scrambled (scr) control vector in Gram-positives
pSelect$_{EfS3}$	F1<br>F2	pSelect$_{T4}$<br>String$_{EfS3\text{-}RSRSR}$	P5<br>P7	P6<br>P8	this study	spacer pair targeting *cps* and of phage EfS3
pSelect$_{EfS7}$	F1<br>F2	pSelect$_{T4}$<br>String$_{EfS7\text{-}RSRSR}$	P5<br>P7	P6<br>P8	this study	spacer pair targeting *cps* of phage EfS7
pEdit$_{EfS3}$	F1<br>F2	pLEB579<br>String$_{EfS3\text{-}nluc}$	P9<br>P11	P10<br>P12	this study	Editing template for site-directed *nluc* insertion downstream of the *cps* gene of phage EfS3
pEdit$_{EfS7}$	F1<br>F2	pLEB579<br>String$_{EfS7\text{-}nluc}$	P13<br>P15	P14<br>P16	this study	Editing template for site-directed *nluc* insertion downstream of the *cps* gene of phage EfS7
pEdit$_{E}$	F1<br>F2	pUC19<br>String$_{E\text{-}nluc}$	P29<br>P31	P30<br>P32	this study	Editing template for site-directed nluc insertion downstream of the *cps* gene of phage E2/E4
pEdit$_{K}$	F1<br>F2	pUC19-Kan^{R}<br>String$_{K\text{-}nluc}$	P33<br>P35	P34<br>P36	this study	Editing template for site-directed *nluc* insertion downstream of the prohead assembly protein gene of phage K1/K4

Table S5: Primer and synthetic DNA string sequences used in this study. Underlined = repeat sequence; italics = spacer sequence; bold = PAM mutation; bold+underlined = protospacer sequence; bold italics = ribosomal binding site; italics underlined = codon-optimized *nluc* sequence.

Nucleotide name	Sequence 5'-3'	Application
P1	GACAGCATCGCCAGTCACTATTCTAGACTCGAGTCGATGTTAAAC	pSelect, cloning
P2	CTGACTGCGTTAGCAATTTGATATCGCGCGCTATTAATCGCAAC	pSelect, cloning
P3	AAATTGCTAACGCAGTCAGG	pSelect, cloning
P4	ATAGTGACTGGCGATGCT	pSelect, cloning
P5	TCAGCTAGACTTCAGTCTTGAAAAG	pSelect, cloning
P6	GACTCCATTCAACATTGCCGA	pSelect, cloning
P7	GCAGTAATACAGGGGCTTTTC	pSelect, cloning
P8	CCTCTTTCTCAAGTTATCATCGG	pSelect, cloning
P9	AGAAAAACCAAAAGCAGTCGATGTTAAACCGTGTGCTCTACG	$pEdit_{EfS3}$, cloning
P10	GATTTGGTAGAACAACGCGCTATTAATCGCAACATCAAACC	$pEdit_{EfS3}$, cloning
P11	GCGATTAATAGCGCGTTGTTCTACCAAATCGCACG	$pEdit_{EfS3}$, cloning
P12	GGTTTAACATCGACTGCTTTTGGTTTTTCTTCTTTTAC	$pEdit_{EfS3}$, cloning
P13	AGTCGATGTTAAACCGTGTGCTCTACG	$pEdit_{EfS7}$, cloning
P14	CGCGCTATTAATCGCAACATCAAACC	$pEdit_{EfS7}$, cloning
P15	GGGGCTTTTATTTTGGTTTGATGTTG	$pEdit_{EfS7}$, cloning
P16	TTATAGTTTTGGTCGTAGAGCACACG	$pEdit_{EfS7}$, cloning
P17	GAGAAATGGAAGTTGAATTAAG	$pSelect_{EfS}$, $pEdit_{EfS}$, verification
P18	GATAATGAACTGTGCTGATTAC	$pSelect_{EfS}$, $pEdit_{EfS}$, verification
P19	ACGATGCAGAATCTGCTCC	EfS3, verification
P20	GGAACAGTTAGACCGTTCC	EfS3, verification
P21	AAATGACTGATAGCTACGAGTG	EfS7, verfification
P22	TTTGCTTCCGTCAGAAGC	EfS7, verfification
P23	GGTCTGTATTTCCCAGAACC	pSelect, verification
P24	AAGGTTCTGGGAAATACAGACCG	pSelect, verification
P25	TAAATACTGAAATAACTAAGGCTCCC	pSelect, verification
P26	AGATAGATTTAATGCTTCATTAGGTACC	pSelect, verification
P27	ATTCAATAGACAATAAGGTCTTAACGC	pSelect, verification
P28	AAATTCGGACAAGCTTATTGCTCG	pSelect, verification
P29	TGCAGCATAAGAAATGCCGGGCCTGGGGTGCCTAATGA	$pEdit_{E}$, cloning
P30	TTAACCCGGATAACGAATTTTAAGCCAGCCCCGACAC	$pEdit_{E}$, cloning
P31	ACTCATTAGGCACCCCAGGCCCGGCATTTCTTATGCTG	$pEdit_{E}$, cloning
P32	CGGGTGTCGGGGCTGGCTTAAAATTCGTTATCCGGGTTAAG	$pEdit_{E}$, cloning
P33	GGTGGATGGTTTAATTCTCGCCTGGGGTGCCTAATGA	$pEdit_{K}$, cloning
P34	GCTGCTTCTAAATACTTTGACTAAGCCAGCCCCGACAC	$pEdit_{K}$, cloning
P35	CACTCATTAGGCACCCCAGGCGAGAATTAAACCATCCACCTC	$pEdit_{K}$, cloning
P36	GGGTGTCGGGGCTGGCTTAGTCAAAGTATTTAGAAGCAGCT	$pEdit_{K}$, cloning
P37	AATAATATTGAAAAAGGAAGAGTATGAGCCATATTCAACGGG	pUC19-KanR, cloning
P38	GAGTAAACTTGGTCTGACAGTTAGAAAAACTCATCGAGCATC	pUC19-KanR, cloning
P39	CGTTGAATATGGCTCATACTCTTCCTTTTTCAATATTATTGAAG	pUC19-KanR, cloning
P40	TGCTCGATGAGTTTTTCTAACTGTCAGACCAAGTTTACTCA	pUC19-KanR, cloning
P41	CATTAATGCAGCTGGCACGA	$pEDIT_{E/K}$, verification
P42	GTCACAGCTTGTCTGTAAGCG	$pEDIT_{E/K}$, verification
P43	GGTAACTTCATTATCGCTTCC	E, verification
P44	TGTGATTTGTTCTCGGTCTAC	E, verification
P45	GTCTTTATCCAAAACGCATTC	K1/K4 verification
P46	CAAACAGTTCAGCCTTAATAC	K1, verification
P47	CAAACAGGTCAGCTTTGATA	K4, verification
$String_{EfS3-RSRSR}$	AACACGCATTGATTTGAGTCAGCTAGGAGGTGACTGAAGTATATTTTAGATGAAGATTATTTCTTAATAACTAAAAATATGGTATAAT<br>ACTCTTAATAAATGCAGTAATACAGGGGCTTTTCAAGACTGAAGTCTAGCTGAGACAAATAGTGCGATTACGAAATTTTTTAGACAAA<br>AATAGTCTACGAGGTTTTAGAGCTATGCTGTTTTGAATGGTCCCAAAAC*AATTCACCTACGAATACGTCAGCAGTTTCA*GTTTTAGAG<br>CTATGCTGTTTTGAATGGTCCCAAAAC*TAAAATCTACAGTATTACAAAATAAAAAAG*GTTTTAGAGCTATGCTGTTTTGAATGGTCCC<br>AAAACTTCAGCACACTGAGACTTGTTGAGTTCCATGTTTTAGAGCTATGCTGTTTTGAATGGACTCCATTCAACATTGCCGATGATAA<br>CTTGAGAAAGAGGGTTAATACCAGCAGTCGGA	spacer pair targeting *cps* and of phage EfS3
$String_{EfS7-RSRSR}$	AGTATATTTTAGATGAAGATTATTTCTTAATAACTAAAAATATGGTATAATACTCTTAATAAATGCAGTAATACAGGGGCTTTTCAAG<br>ACTGAAGTCTAGCTGAGACAAATAGTGCGATTACGAAATTTTTTAGACAAAAATAGTCTACGAGGTTTTAGAGCTATGCTGTTTTGAA<br>TGGTCCCAAAAC*AACGTGCAATCTTCATCAACAAGGTAGCTG*GTTTTAGAGCTATGCTGTTTTGAATGGTCCCAAAAC*AGACTTATAA*<br>*TATTTACCAAGATGGAGCGA*GTTTTAGAGCTATGCTGTTTTGAATGGTCCCAAAACTTCAGCACACTGAGACTTGTTGAGTTCCATGT<br>TTTAGAGCTATGCTGTTTTGAATGGACTCCATTCAACATTGCCGATGATAACTTGAGAAAGAGGGTTAATACCAGCAGTCGGATACCT<br>TCCTATTCTTTCTGTTAAAGCGTTTTCATGTTATAATAGGCAAAAGAAGAGTAGTGTGATCGTCCATTCCGACAGCATCGCCAGTCAC<br>TAT	spacer pair targeting *cps* of phage EfS7
$String_{T4-RSRSR}$	AACACGCATTGATTTGAGTCAGCTAGGAGGTGACTGAAGTATATTTTAGATGAAGATTATTTCTTAATAACTAAAAATATGGTATAAT<br>ACTCTTAATAAATGCAGTAATACAGGGGCTTTTCAAGACTGAAGTCTAGCTGAGACAAATAGTGCGATTACGAAATTTTTTAGACAAA<br>AATAGTCTACGAGGTTTTAGAGCTATGCTGTTTTGAATGGTCCCAAAAC*TATACACGTCTAAAGTAAGCGTTTTTACCA*GTTTTAGAG<br>CTATGCTGTTTTGAATGGTCCCAAAAC*GTCCTAAAACTATTCGCAATTGGATAAATG*GTTTTAGAGCTATGCTGTTTTGAATGGTCCC<br>AAAACTTCAGCACACTGAGACTTGTTGAGTTCCATGTTTTAGAGCTATGCTGTTTTGAATGGACTCCATTCAACATTGCCGATGATAA<br>CTTGAGAAAGAGGGTTAATACCAGCAGTCGGA	spacer pair targeting *cps* of phage T4
$String_{E-RSRSR}$	AACACGCATTGATTTGAGTCAGCTAGGAGGTGACTGAAGTATATTTTAGATGAAGATTATTTCTTAATAACTAAAAATATGGTATAAT<br>ACTCTTAATAAATGCAGTAATACAGGGGCTTTTCAAGACTGAAGTCTAGCTGAGACAAATAGTGCGATTACGAAATTTTTTAGACAAA<br>AATAGTCTACGAGGTTTTAGAGCTATGCTGTTTTGAATGGTCCCAAAAC*ACCCATTTGCAGAATCCGCTGCTCAGGCTC*GTTTTAGAG<br>CTATGCTGTTTTGAATGGTCCCAAAAC*TTGGGCGACCGATTGAGTTGCTATTCGTTG*GTTTTAGAGCTATGCTGTTTTGAATGGTCCC<br>AAAACTTCAGCACACTGAGACTTGTTGAGTTCCATGTTTTAGAGCTATGCTGTTTTGAATGGACTCCATTCAACATTGCCGATGATAA<br>CTTGAGAAAGAGGGTTAATACCAGCAGTCGGA	spacer pair targeting *cps* and *gp170* of phage E2/E4

String$_{K\text{-}CPS\text{-}RSRSR}$	AACACGCATTGATTTGAGTCAGCTAGGAGGTGACTGAAGTATATTTTAGATGAAGATTATTTCTTAATAACTAAAAATATGGTATAAT<br>ACTCTTAATAAATGCAGTAATACAGGGGCTTTTCAAGACTGAAGTCTAGCTGAGACAAATAGTGCGATTACGAAATTTTTTAGACAAA<br>AATAGTCTACGAGGTTTTAGAGCTATGCTGTTTTGAATGGTCCCAAAAC*CGAGAGCCATAAAAGCATCATCAGCACTAT*GTTTTAGAG<br>CTATGCTGTTTTGAATGGTCCCAAAAC*GAGCGACGGAAGCATCGATAGTTTTAGCTT*GTTTTAGAGCTATGCTGTTTTGAATGGTCCC<br>AAAACTTCAGCACACTGAGACTTGTTGAGTTCCATGTTTTAGAGCTATGCTGTTTTGAATGGACTCCATTCAACATTGCCGATGATAA<br>CTTGAGAAAGAGGGTTAATACCAGCAGTCGGA	spacer pair targeting *gp167* and *gp168* of phage K1 (= spacer pair targeting *gp168* and *gp169* of phage K4)
String$_{EfS3\text{-}nluc}$	ATTCTGCAATCATTAACGCTACTGACGGTGTTAAATTAGCAATCACTATCAACAGCATGTATCAAGCTCGTCCTCAATACGTAGCTAT<br>CTACCGTAAAGGACATCAAACAGGATTGTTCTACCAAATCGCACGTGTACCTGCAAGTAAAGCAGAAAACAACGTAATCACTTTCTAC<br>GATTTGAACCAACAAAT**T**CC**TGAAACTGCTGACGTATTCGTAGGTGAATT**GACTCCATCTGTAGTTCACTTGTTCGAATTACTACCAA<br>TGATGCGTTTACCATTAGCTCAAATGAACGCTTCTGTAACATTCGCAGTTCTATGGTATGGTGCTTTAGCTCTTCGTGCACCTAAGAA<br>ATGGGCACGAATCAAGAACGTTAAATACATTCCATCTGGAAACGTAATTAACGAACGCTAA***GAGGAGGTAAATATAT*** *ATGGTTTTCAC*<br>*ATTAGAAGATTTCGTTGGTGATTGGCGTCAAACAGCTGGTTACAACTTAGATCAAGTTTTAGAACAAGGTGGTGTTTCATCATTATTC*<br>*CAAAACTTAGGTGTTTCAGTTACACCAATCCAACGTATCGTTTTATCAGGTGAAAACGGTTTAAAAATCGATATCCACGTTATCATCC*<br>*CATACGAAGGTTTATCAGGTGATCAAATGGGTCAAATCGAAAAAATCTTCAAAGTTGTTTACCCAGTTGATGATCACCACTTCAAAGT*<br>*TATCTTACACTACGGTACATTAGTTATCGATGGTGTTACACCAAACATGATCGATTACTTCGGTCGTCCATACGAAGGTATCGCTGTT*<br>*TTCGATGGTAAAAAAATCACAGTTACAGGTACATTATGGAACGGTAACAAAATCATCGATGAACGTTTAATCAACCCAGATGGTTCAT*<br>*TATTATTCCGTGTTACAATCAACGGTGTTACAGGTTGGCGTTTATGTGAACGTATCTTAGCTTAA* TCTTAGTTCAACTAAGAAAAATT<br>GAATAGAAAACGGAATAGGGAACGGTCTAACTGTTCCCTATTTTTATTATAACGAATTAAATGGAGGAATACAAATGT**TAAAATCTAC**<br>**AGTATTACAAAATAAAAAAG**T**T**GCAACATCTTTTGGAGAAGCTGAATTTAACCATTTAGGAGAAAGTCATAACTTGCCAGAGGAACAT<br>CAAAAAGCTTTAGCTGAAAAGGTACCTTATTTGACACACATTCCTGATAAGAAAGAAAATCCTAAAGAGGAAAAAGAAGTAAAAGAAG<br>AAAAACCAAAAGCTAAAAAAGCACCTGCTAAAGCTAAAACAGCCAAAAAAGATAAAGAGTAGGAGGTCATCATATGTACCCAGATTAC<br>GGTTATGGTGATGGCGGAAACCAAGTTAACCGTGAATAT	Editing template for site-directed *nluc* insertion downstream of major capsid protein (*cps*) sequence of phage EfS3
String$_{EfS7\text{-}nluc}$	AAAGTCGAAGGGGGCTTTTATTTTGGTTTGATGTTGCGATTAATAGCGCGTGAAATGACTGATAGCTACGAGTGGGTTATTGATGCTC<br>AAGTAGCATTTGGCGAAGAGTTTGATGAAGAAACTTACTTATTCATCTCACCTAAACGCCGTGCAACAATTTTGAAATCTAAAGACTT<br>CATTCACATTCAACAAGGCGTGTCTGTAATCAAAGGCCACTTAGGTGAAATCTACGGAATGAACATTGTAGTTTCTAACAAGGTTGGT<br>GAAGACGAAGCGTTCGTATTAAAACGTGGAGCTTTATCATTAATTATGAAACGTGACTACATGGTTGAAGAAGTGCGTGAAGGTATGA<br>AACGTCAAACTAACATCACTGCTGACCAACACTACGTAGCATTCGTTAAAGATGCTA**AACGTGCAATCTTCATCAACAAGGTAGCTG**C<br>**A**GGAAAGTAG***GAGGAGGTAAATATAT*** *ATGGTTTTCACATTAGAAGATTTCGTTGGTGATTGGCGTCAAACAGCTGGTTACAACTTAGA*<br>*TCAAGTTTTAGAACAAGGTGGTGTTTCATCATTATTCCAAAACTTAGGTGTTTCAGTTACACCAATCCAACGTATCGTTTTATCAGGT*<br>*GAAAACGGTTTAAAAATCGATATCCACGTTATCATCCCATACGAAGGTTTATCAGGTGATCAAATGGGTCAAATCGAAAAAATCTTCA*<br>*AAGTTGTTTACCCAGTTGATGATCACCACTTCAAAGTTATCTTACACTACGGTACATTAGTTATCGATGGTGTTACACCAAACATGAT*<br>*CGATTACTTCGGTCGTCCATACGAAGGTATCGCTGTTTTCGATGGTAAAAAAATCACAGTTACAGGTACATTATGGAACGGTAACAAA*<br>*ATCATCGATGAACGTTTAATCAACCCAGATGGTTCATTATTATTCCGTGTTACAATCAACGGTGTTACAGGTTGGCGTTTATGTGAAC*<br>*GTATCTTAGCTTAA* CAGCCCCCAAGAATCTTCAATCTAGCGGGGTTACTGAGGACTCAGTTACTCTGACTTGGGAAGACGGAGGGGCA<br>AGCCTCTAGTAAGCTAAACGAGAGGTTGCGGAAACGTTCCGTGACCTCTTTTACTTTATCATAAATCAGGAGGAAAAGCAATGGCAA**A**<br>**GACTTATAATATTTACCAAGATGGAGCGA**A**A**GTTCAGGAAGGCATTGCAGAGTTAACTAAAACCATTACAGGCCTTACGCCAAACACT<br>TCATACAAGTTTGAGGTAACAGCTGTTGAGGACGGCGTGGAGAGTGCTAAGTCCACAGCGGTAACAGCCAAAACAAACCCGCGTAAAG<br>TAGCTACAGTAACAGCTTCTCAAAAGACTATGTCTCTAGCTTCTGACGGAAGCAAAGCATTGAGTCGATGTTAAACCGTGTGCTCTAC<br>GACCAAAACTATAAAACCTTTAAG	Editing template for site-directed *nluc* insertion downstream of major capsid protein (*cps)* sequence of phage EfS7
String$_{E\text{-}nluc}$	CCGGCATTTCTTATGCTGCACAAGGTCTGGCTACTGGCTTTAACACTGATACTACCAAGTCAGTATTTGCTGGTGTTCTGGGTGGTAA<br>ATACCGTGTATATATCGACCAGTATGCTAAACAGGATTATTTCACCGTAGGTTATAAAGGTCCGAACGAAATGGATGCTGGTATTTAC<br>TATGCTCCATATGTAGCTCTGACTCCGCTGCGTGGTTCTGATCCGAAGAACTTCCAACCGGTAATGGGATTCAAAACTCGTTACGGTA<br>TCGGTATCA**ACCCATTTGCAGAATCCGCTGCTCAGGCTC**C**A**GCTTCTCGCATCCAGAGCGGTATGCCTTCTATTCTGAATAGCCTTGG<br>TAAAAACGCTTACTTCCGCCGTGTATATGTTAAAGGTATCTAAA ***GTACGAGGAGGTAAATATAT*** *ATGGTTTTCACCCTGGAAGACTT*<br>*CGTTGGTGACTGGCGTCAGACCGCTGGTTACAACCTCGACCAGGTGCTAGAACAGGGTGGTGTTTCTTCTTTATTCCAGAACCTGGGT*<br>*GTAAGCGTTACCCCGATCCAGCGTATCGTTCTGTCTGGTGAAAACGGTCTGAAAATCGACATCCACGTTATCATCCCGTACGAAGGTC*<br>*TGTCTGGTGACCAGATGGGTCAGATCGAAAAAATCTTCAAAGTTGTTTACCCGGTTGACGACCACCACTTCAAAGTTATCCTGCACTA*<br>*CGGTACCCTGGTTATCGACGGTGTTACCCCGAACATGATCGACTACTTCGGTCGTCCGTACGAAGGTATCGCTGTTTTCGACGGTAAA*<br>*AAAATCACCGTTACCGGTACCCTGTGGAACGGTAACAAAATCATCGACGAACGTCTGATCAACCCGGACGGTTCTCTGCTGTTCCGTG*<br>*TTACCATCAACGGTGTTACCGGTTGGCGTCTGTGCGAACGTATCCTGGCTTAA* TCTTTGACGATTTAAGGGACCTTCGGGTCCCTTTA<br>GTTGTTTTTATGGTATAAATAAATCATATAAACTGAAAGGAAAGCGCAATGGCTAAAATCAACGAACTTCTGCGCGAATCAAC**A**A**CAA**<br>**CGAATAGCAACTCAATCGGTCGCCCAA**ATCTCGTTGCTTTGACTCGCGCTACCACTAAATTAATATATTCTGACATTGTAGCAACGCA<br>AAGAACTAATCAACCTGTTGCTGCTTTTTATGGTATCAAATACCTTAACCCGGATAACGAATTT	Editing template for site-directed *nluc* insertion downstream of the *cps* gene of phage E2/E4
String$_{K\text{--}nluc}$	GAGAATTAAACCATCCACCTCGTGCAAATGTTGACCCGATGCAAGCCGCAATCATTATCGAAGATATGTGGTGGAAAGGCAACGATGT<br>TTATGGGCGTGCAAGAATCATCGAAGGAGACCACGGTCCAGGTGATAAATTAGCCGCTAACATCCGTGCTGGTTGGATTCCTGGTGTA<br>TCAAGCCGTGGGCTCGGTTCTTTAAAAGACTCAGGAAAAGGATATAACATCGTACAAGAAGGCTTTCGCCTGACTGTTGGTGTAGACG<br>CAGTTTGGGGTCCTAGTGCTCCGGATGCATGGGTACAACCGAAACAAATTTCGGAAAATACCTCGGCACAAGTTGC**A**A**ATAGTGCTGA**<br>**TGATGCTTTTATGGCTCTCG**CAGAGAAGTTAAAAGCGTTATAA*AGTAC* ***GAGGAGGTAAATATAT*** *ATGGTTTTCACCCTGGAAGACTT*<br>*CGTTGGTGACTGGCGTCAGACCGCTGGTTACAACCTCGACCAGGTGCTAGAACAGGGTGGTGTTTCTTCTTTATTCCAGAACCTGGGT*<br>*GTAAGCGTTACCCCGATCCAGCGTATCGTTCTGTCTGGTGAAAACGGTCTGAAAATCGACATCCACGTTATCATCCCGTACGAAGGTC*<br>*TGTCTGGTGACCAGATGGGTCAGATCGAAAAAATCTTCAAAGTTGTTTACCCGGTTGACGACCACCACTTCAAAGTTATCCTGCACTA*<br>*CGGTACCCTGGTTATCGACGGTGTTACCCCGAACATGATCGACTACTTCGGTCGTCCGTACGAAGGTATCGCTGTTTTCGACGGTAAA*<br>*AAAATCACCGTTACCGGTACCCTGTGGAACGGTAACAAAATCATCGACGAACGTCTGATCAACCCGGACGGTTCTCTGCTGTTCCGTG*<br>*TTACCATCAACGGTGTTACCGGTTGGCGTCTGTGCGAACGTATCCTGGCTTAA* ATAATTATGTAACTTAACACAGGACATCAAAAATG<br>CTTAAAGAACTTTTAATGAC**A**G**AAGCTAAAACTATCGATGCTTCCGTCGCTC**TTGACAGTATTTTCGAATCAGTTCAGCTTTCTCCGG<br>AAGCTAAAGCTAACTTCAGCACTGTATTCGAAGCAACAGTCAAAAAACACGCCGTAGCTCTGGCAGAATCTCACATTGAAAAGATTGC<br>TGAGAAAGCAGAAGAAAAAGTAGAAGAAGAAAAAGAAAAAGCTAAAGAAGAAGCTCGCGAAGAGCTGAAAGAAGCTGCTTCTAAATAC<br>TTTGAC	Editing template for site-directed *nluc* insertion downstream of the prohead assembly protein gene of phage K1/K4
