# Supplementary material for: Engineered reporter phages for detection of Escherichia coli, Enterococcus, and Klebsiella in urine
Source: Nat Commun. 2023 Jul 20;14:4336. doi: 10.1038/s41467-023-39863-x (PMC10359277; doi:10.1038/s41467-023-39863-x)
Supplement: Supplementary file 3 — Reporting Summary [file 41467_2023_39863_MOESM3_ESM.pdf]

nature portfolio

Corresponding author(s): Samuel Kilcher

Last updated by author(s): Jun 21, 2023

Reporting Summary

Nature Portfolio wishes to improve the reproducibility of the work that we publish. This form provides structure for consistency and transparency in reporting. For further information on Nature Portfolio policies, see our Editorial Policies and the Editorial Policy Checklist.

Statistics

For all statistical analyses, confirm that the following items are present in the figure legend, table legend, main text, or Methods section.

n/a	Confirmed	
☐	☒	The exact sample size (*n*) for each experimental group/condition, given as a discrete number and unit of measurement
☐	☒	A statement on whether measurements were taken from distinct samples or whether the same sample was measured repeatedly
☒	☐	The statistical test(s) used AND whether they are one- or two-sided<br>*Only common tests should be described solely by name; describe more complex techniques in the Methods section.*
☒	☐	A description of all covariates tested
☒	☐	A description of any assumptions or corrections, such as tests of normality and adjustment for multiple comparisons
☐	☒	A full description of the statistical parameters including central tendency (e.g. means) or other basic estimates (e.g. regression coefficient) AND variation (e.g. standard deviation) or associated estimates of uncertainty (e.g. confidence intervals)
☒	☐	For null hypothesis testing, the test statistic (e.g. *F*, *t*, *r*) with confidence intervals, effect sizes, degrees of freedom and *P* value noted<br>*Give P values as exact values whenever suitable.*
☒	☐	For Bayesian analysis, information on the choice of priors and Markov chain Monte Carlo settings
☒	☐	For hierarchical and complex designs, identification of the appropriate level for tests and full reporting of outcomes
☒	☐	Estimates of effect sizes (e.g. Cohen's *d*, Pearson's *r*), indicating how they were calculated

Our web collection on statistics for biologists contains articles on many of the points above.

Software and code

Policy information about availability of computer code

Data collection	Spectrostar Omega MARS Data Analysis Software (MARS version: 3.42 R6)<br>GloMax® Navigator Luminometer (Promega) (internal software)
Data analysis	GraphPad Prism (Version 9)<br>CLC Genomics Workbench (Version 20)<br>RAST server (https://rast.nmpdr.org/)<br>tRNAscan-SE version 2 (http://lowelab.ucsc.edu/tRNAscan-SE/)<br>MLST and SeroTypeFinder (www.genomicepidemiology.org/)<br>MALDI Biotyper software package (MBT Compass 4.1)

For manuscripts utilizing custom algorithms or software that are central to the research but not yet described in published literature, software must be made available to editors and reviewers. We strongly encourage code deposition in a community repository (e.g. GitHub). See the Nature Portfolio guidelines for submitting code & software for further information.

Data

Policy information about availability of data

All manuscripts must include a data availability statement. This statement should provide the following information, where applicable:

- Accession codes, unique identifiers, or web links for publicly available datasets
- A description of any restrictions on data availability
- For clinical datasets or third party data, please ensure that the statement adheres to our policy

"Phage genomes are available from the GenBank database: E2 (UTI-E2; OL870316), E4 (UTI-E4; OL870317), K1 (UTI-K1; OL870318), K4 (UTI-K4; OL870319), EfS3 (UTI-EfS3; OL870611), and EfS7 (UTI-EfS7; OL870612). All other data that support the findings of this study are available from the Source Data File."

Human research participants

Policy information about studies involving human research participants and Sex and Gender in Research.

Reporting on sex and gender	For this study, biological material (i.e., urine and bacterial strains) was collected from female and male patients (sex, assigned by urologist).<br>For the Zurich Uropathogen Collection, bacterial strains isolated from urine samples from 307 patients were analyzed and the sex distribution was 46.9 % female (144/307) and 53.1 % male (163/307). For the field evaluation, urine from 206 patients was analyzed and the sex distribution was 35.15 % female (71/202) and 64.85 % male (131/202). Sex information was missing for 4 samples.
Population characteristics	We do not analyze covariate population characteristics in this study. Patients from both sex, ≥18 years old and treated at the tertiary neuro-urology department (Balgrist University Hospital, University of Zürich, Zürich, Switzerland) were considered eligible for the Zurich Uropathogen Collection and for the field evaluation. For the Zurich Uropathogen collection, the age mean (Q1-Q3) was 60 (51-75). For the field evaluation, which also included samples from the department of urology (University Hospital Zurich, University of Zurich, Zurich, Switzerland), the age mean (Q1-Q3) was 60 (49-69).
Recruitment	For the Zurich Uropathogen Collection patients were recruited from the in- and outpatient clinic of the Department of Neuro-Urology, Balgrist University Hospital, University of Zurich, Zurich, Switzerland from January to December 2020 in the in a consecutive manner. If a urine culture was indicated as part of a patient's treatment (i.e., for prophylactic reasons prior to invasive diagnostics and/or surgery or due to UTI symptoms) then the patient was asked to provide written informed consent to reuse health-related personal data and their biological material (urine sample) for research purposes. Urine cultures were grown at the Institute of Medical Microbiology (University of Zurich, Switzerland) and subsequently provided to ETH Zurich for integration into the Zurich Uropathogen Collection.<br>To assess the reporter phage-based detection of the target pathogen (field evaluation), patients were recruited at the Balgrist University Hospital and at the University Hospital Zurich (Department of urology, University of Zurich, Zurich, Switzerland) from February to June 2021. Whenever urine culture was indicated for routine microbiological diagnostic analysis, informed consent was provided as described above and an additional urine sample was collected and provided to ETH for selective/differential plating and to assess reporter phage-based diagnostic.
Ethics oversight	As described in the main text, for the collection of patient urine specimens: "All patients gave a general written informed consent, in line with the local ethics committee (Kantonale Ethikkommission Zurich, Switzerland), agreeing for further use of health-related personal data and biological material for research purposes. The study was performed in accordance with the World Medical Association Declaration of Helsinki (41) and conformed with the International Conference on Harmonisation (ICH) Good Clinical Practice (GCP) Guidelines (E6) and the International OrganizationAll patients gave a general written informed consent, in line with the local ethics committee (Kantonale Ethikkommission Zurich, Switzerland), agreeing for further use of health-related personal data and biological material for research purposes. The study was performed in accordance with the World Medical Association Declaration of Helsinki (41) and conformed with the International Conference on Harmonisation (ICH) Good Clinical Practice (GCP) Guidelines (E6) and the International Organization for Standardization (ISO, 14,155). for Standardization (ISO, 14,155)."

Note that full information on the approval of the study protocol must also be provided in the manuscript.

Field-specific reporting

Please select the one below that is the best fit for your research. If you are not sure, read the appropriate sections before making your selection.

☒ Life sciences ☐ Behavioural & social sciences ☐ Ecological, evolutionary & environmental sciences

For a reference copy of the document with all sections, see nature.com/documents/nr-reporting-summary-flat.pdf

Life sciences study design

All studies must disclose on these points even when the disclosure is negative.

Sample size	No statistical method was performed to predetermine sample sizes for our experiments. Instead, the sample sizes listed below were chosen based on practical considerations, previous similar studies in the field, and the specific objectives of each experiment.<br><br>Samples sizes and replicates (biological or technical) are listed below for all experiments performed in this study:<br><br>Fig. 1a: Sample size is not applicable.<br>Fig. 1b: A single representative transmission electron microscopy image of each phage (Fig. 1b) was selected from multiple micrographs taken from a single phage-coated grid as is common practice.<br><br>Fig. 2b/c: Representative spot-on-the-lawn images are shown for data quantified in Fig. S2b-c (which were performed as biological triplicates, see below).<br>Fig. 2d: 10 plaques were isolated from one replicate of data shown in Fig. S2b-c.<br><br>Fig. 3a: Representative plaque morphologies are shown from a single replicate of soft-agar overlays performed with the indicated phages.<br>Fig. 3b: Infection kinetics of engineered vs. wildtype phages were determined using the liquid infection assay as technical triplicates. Mean +/- standard deviation is shown.<br>Fig. 3c: Bioluminescence time-course assays were performed as biological triplicates. Mean +/- standard error of the mean (SEM) is shown.<br>Fig. 3d: Datapoints used for linear regression and background luminescence are from biological triplicates.<br><br>Fig. 4a: Plaque host ranges and bioluminescence detection ranges were determined from single infection / bioluminescence assays.<br>Fig. 4c-e: Data is from biological triplicates. Means are shown, error bars are SEM.<br><br>Fig. 5: All data is from patient material and was measured once.<br><br>Fig. 6b-d: All in vitro data is shown as mean ± standard deviation from technical triplicates. Luminescence values were determined as single measurements from fresh patient urine.<br><br>Fig. S1: We collected all urological isolates from the Department of Neuro-urology at the Balgrist University Hospital (Zurich, Switzerland) for a full year (2020), which should reflect the local UTI etiology (227 incidents of UTI and 215 incidents of asymptomatic bacteriuria).<br><br>Fig. S2b-c: Quantification of plaquing efficiency was performed using the soft-agar overlay method as biological triplicates. Data is mean +/- SEM.<br><br>Fig. S3a-f: All luminescence data is shown as the mean from biological triplicates.<br>Fig. S3g: Antibiotic susceptibility testing was performed at the IMM according to the EUCAST guidelines (Version 11.0, 2021, www.eucast.org).<br><br>Fig. S4: 274 bacterial strains were isolated from 206 patient urine samples collected from February to June 2021.<br><br>Fig. S5: All experiments were performed as biological triplicates with the mean shown.
Data exclusions	No data was excluded.
Replication	All in vitro assays were performed as biological or technical triplicates (as described above) with the exception of the field evaluation. Due to time and material limitations of working with multiple fresh urine samples, the raw luminescence values measured during the field evaluation (Fig. 5) were measured once. From previous experiments in urine (see Fig. 4d) we knew that bioluminescence is highly reproducible.
Randomization	There was no requirement for randomization as all samples and data points in this study were analyzed and reported equally with no sub-sampling. For instance, the urine samples analyzed in Fig. 5 were simply collected consecutively throughout sampling days, without any pre-selection based on age, sex, gender, or treatment plan. In the lab, these samples were all tested and reported the same way with no sample exclusion.
Blinding	Blinding was not required in this study as all samples and data points were tested and analyzed equally with no sub-sampling. Furthermore, all urine samples were tested blind to any prior microbiological identification as results from standard-of-care diagnostics were not available at the time of conducting the reporter phage analysis.

Reporting for specific materials, systems and methods

We require information from authors about some types of materials, experimental systems and methods used in many studies. Here, indicate whether each material, system or method listed is relevant to your study. If you are not sure if a list item applies to your research, read the appropriate section before selecting a response.

Materials & experimental systems

n/a	Involved in the study
☒	☐ Antibodies
☒	☐ Eukaryotic cell lines
☒	☐ Palaeontology and archaeology
☒	☐ Animals and other organisms
☒	☐ Clinical data
☒	☐ Dual use research of concern

Methods

n/a	Involved in the study
☒	☐ ChIP-seq
☒	☐ Flow cytometry
☒	☐ MRI-based neuroimaging
